# A Bayesian approach for estimating the probability of trigger failures in the stop-signal paradigm

**DOI:** 10.3758/s13428-015-0695-8

**Published:** 2016-01-28

**Authors:** Dora Matzke, Jonathon Love, Andrew Heathcote

**Affiliations:** 1 Department of Psychology, University of Amsterdam, Weesperplein 4, 1018 XA Amsterdam, The Netherlands; 2 School of Medicine, Division of Psychology, University of Tasmania, Tasmania, Australia; 3 School of Psychology, University of Newcastle, Callaghan, NSW Australia

**Keywords:** Bayesian hierarchical modeling, Ex-Gaussian distribution, Response inhibition, Stop-signal paradigm, Stop-signal RT distribution, Trigger failure

## Abstract

Response inhibition is frequently investigated using the stop-signal paradigm, where participants perform a two-choice response time task that is occasionally interrupted by a stop signal instructing them to withhold their response. Stop-signal performance is formalized as a race between a go and a stop process. If the go process wins, the response is executed; if the stop process wins, the response is inhibited. Successful inhibition requires fast stop responses and a high probability of triggering the stop process. Existing methods allow for the estimation of the latency of the stop response, but are unable to identify deficiencies in triggering the stop process. We introduce a Bayesian model that addresses this limitation and enables researchers to simultaneously estimate the probability of trigger failures and the entire distribution of stopping latencies. We demonstrate that trigger failures are clearly present in two previous studies, and that ignoring them distorts estimates of stopping latencies. The parameter estimation routine is implemented in the BEESTS software (Matzke et al., *Front. Quantitative Psych. Measurement, 4*, 918; 2013a) and is available at http://dora.erbe-matzke.com/software.html.

**Electronic supplementary material** The online version of this article (doi:10.3758/s13428-015-0695-8) contains supplementary material, which is available to authorized users.

## Introduction

Response inhibition refers to the ability to stop an ongoing response that is no longer appropriate, such as rapidly stopping when a traffic light turns red. Inhibition is the hallmark of executive functions and has received—and continues to receive—considerable attention in psychology. Response inhibition is frequently investigated with the stop-signal paradigm (Logan and Cowan 1984). Over the past 35 years, the stop-signal paradigm has facilitated the interpretation of numerous developmental, experimental, and neuropsychological studies (e.g., Bissett and Logan, 2011; Forstmann et al., 2012; Williams et al., 1999), and has been applied to examine the nature of inhibition deficits in clinical conditions, such as schizophrenia (Badcock et al. 2002; Hughes et al. 2012) and attention deficit hyperactivity disorder (e.g., ADHD, Schachar and Logan, 1990).

In the stop-signal paradigm, participants perform a two-choice response time (RT) task. This primary task is occasionally interrupted by a stop signal that instructs participants to withhold their choice response. Response inhibition can be conceptualized as a race between two independent processes: a go process that is initialized by the primary (choice-task) stimulus and a stop process that is triggered by the stop signal. If the go process wins, a response is executed; if the stop process wins, the response is inhibited (Logan and Cowan 1984). The race model allows for the estimation of the unobservable latency of the stop response (stop-signal reaction time [SSRT]). Successful response inhibition, however, not only requires relatively fast stop responses, but the stop process must also be successfully triggered before it can begin the race against the go process; if participants fail to encode and correctly interpret the stop signal, they cannot even attempt to stop the ongoing response.

Trigger failures pose well-known theoretical and methodological challenges to the interpretation of stop-signal data (Logan 1994). First, differences in inhibition performance across groups may result from differences in the latency or variability of the stop response, but they might just as well reflect differences in the probability of triggering the stop process. For instance, poor response inhibition in ADHD and schizophrenia may result from a slower or more variable stop response, but it may also reflect a stop process that is not triggered reliably (e.g., Badcock et al., 2002; Schachar and Logan, 1990).

Second, trigger failures can bias the estimation of stopping latencies. Band et al. (2003) have shown that trigger failures can result in a dramatic overestimation of SSRTs. As we will demonstrate shortly, trigger failures can also bias the estimation of entire SSRT distributions. As a result, trigger failures may cause fictitious group differences in estimated SSRT; researchers may mistakenly conclude that two groups differ in the speed of the stop process because of undetected differences in the probability of trigger failures. They may also conclude that no difference in inhibition exists when trigger failure and the speed of stopping differ in opposite directions.

### Previous attempts to identify trigger failures

Previous attempts to identify trigger failures have been based on inhibition functions. Inhibition functions describe the relationship between signal-respond rate (i.e., P(response ∣ stop signal)) and the interval between the onset of the primary task stimulus and the stop signal (stop-signal delay [SSD]). SSDs are typically set according to the fixed-SSD or the staircase-tracking procedure (Logan 1994). With the fixed-SSD procedure, participants are presented with stop signals at a number of a priori chosen SSDs. With staircase tracking, SSDs are set dynamically contingent on participants’ performance, with the aim of achieving an overall signal-respond rate of 0.50.

**Fig. 1:**
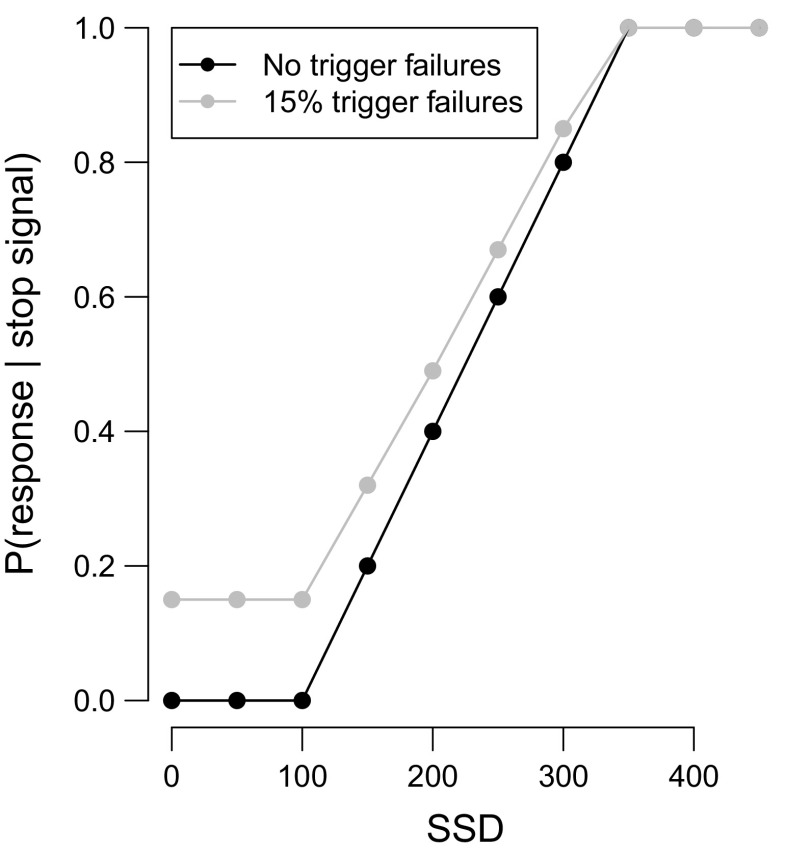
Inhibition function with and without trigger failures. SSD = stop-signal delay

Regardless of the SSD procedure, the race model predicts that signal-respond rate increases with increasing SSD. The black line in Fig. 1 shows an inhibition function for the fixed-SSD procedure with a stop process that is reliability triggered on every stop-signal trial. The inhibition function asymptotes at 0 for short SSDs and increases steeply with increasing SSD. In contrast, the gray line shows an inhibition function for a stop process with a trigger-failure probability of 15 %. This inhibition function is less steep and asymptotes around 0.15, instead of 0, for early SSDs.

Unfortunately, in practice, inhibition functions cannot be used to identify trigger failures. First, differences in inhibition functions not only reflect differences in the probability of trigger failures but also differences in the latency and variability of the go and the stop process. Logan and Cowan (1984) have suggested correcting inhibition functions for differences in primary task “go” RTs, SSRTs, and go RT variability using the ZRFT transformation. However, ZRFT-transformed inhibition functions are unable to discriminate between the effects of SSRT variability and trigger failures, and also fail to adequately account for differences in go RT variability (Band et al. 2003). Second, although the lower asymptote of the inhibition function can theoretically provide an indication of the probability of trigger failures, in practice, obtaining a sufficiently stable lower tail estimate may require an impractically large number of stop-signal trials. This approach is also incompatible with the widely used tracking procedure, which typically yields only a few stop-signal trials at short SSDs.

Despite its theoretical and methodological importance, the problem of quantifying the contribution of trigger failures to stop-signal performance is presently unsolved. We address this limitation and describe a Bayesian method that allows researchers to reliably estimate the probability of trigger failures as well as the entire distribution of SSRTs. We first outline our approach and introduce the basic concepts of Bayesian parameter estimation. We then investigate its performance in two simulation studies. In the first, we assess the asymptotic performance of the model and show that—in contrast to other methods—it accurately recovers SSRTs even in the presence of relatively frequent trigger failures. In the second, we investigate the number of observations that are necessary for accurate parameter estimation. Lastly, we illustrate the advantages of our trigger-failure framework with two published stop-signal data sets.

## Methods

### Simultaneous estimation of SSRT distributions and trigger failures

**Fig. 2:**
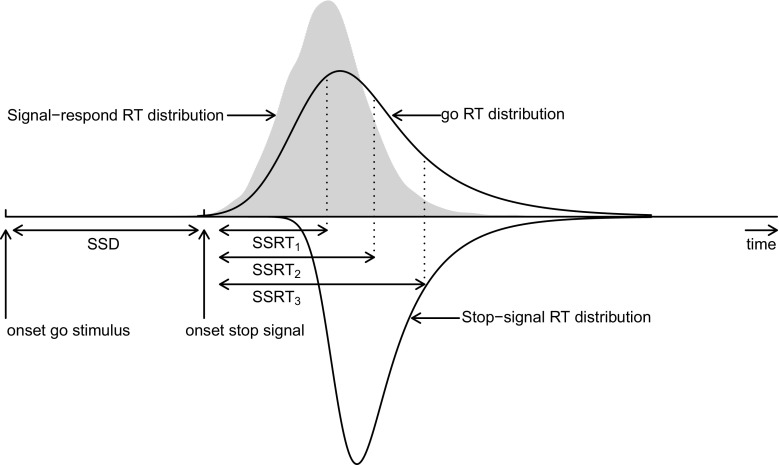
The race model. go RT = primary task RT; SSD = stop-signal delay; SSRT = stop-signal reaction time. The model assumes that go RTs and SSRTs are random variables. BEESTS treats the distribution of signal-respond RTs (*gray area*) as a go RT distribution that is censored by the SSRT distribution. The censoring point can take on a different value on each stop-signal trial (e.g., *SSD* + *SSRT*_1_, *SSD* + *SSRT*_2_, and *SSD* + *SSRT*_3_; see also Matzke et al., 2013b)

The estimation of SSRT distributions follows the Bayesian parametric—BEESTS—approach developed by Matzke et al. (2013a, b). As shown in Fig. 2, BEESTS is based on a race model that treats both go RTs and SSRTs as random variables. If the go RT is slower than *S* *S* *D* + *S* *S* *R* *T* on a given trial, the go RT is inhibited. If the go RT is faster than *S* *S* *D* + *S* *S* *R* *T*, the go RT cannot be inhibited and results in a signal-respond RT. BEESTS treats the signal-respond (i.e., failed inhibition) RT distribution as a censored go RT distribution. The censoring point is randomly drawn from the SSRT distribution on each stop-signal trial. Estimation of the SSRT distribution involves simultaneously estimating the go RT distribution and its censoring distribution.

BEESTS assumes that go RTs and SSRTs follow an ex-Gaussian distribution. The ex-Gauss is a commonly used description of RT distributions obtained by adding a Gaussian and an exponential random variable (Heathcote et al. 1991; Matzke and Wagenmakers 2009). The ex-Gauss has three parameters: *μ* and *σ* reflect the leading edge of the distribution, and *τ* reflects its slow tail.

#### Estimating trigger failures

We augment the standard BEESTS model with a parameter *P*(*T* *F*) that quantifies the probability of trigger failures. The resulting mixture model assumes that signal-respond RTs are produced with (1) probability *P*(*T* *F*) if the stop process was not triggered; or (2) probability 1−*P*(*T* *F*) if the stop process was successfully triggered but has finished after the go process (i.e., go RT <*S* *S* *D* + *S* *S* *R* *T*). Formally, the likelihood (*L*_*SR*_) of the *r* = 1,..., *R* signal-respond RTs for a given SSD is:

1 $$\begin{array}{@{}rcl@{}} &&\L_{SR}(\mu_{go}, \sigma_{go},\tau_{go},\mu_{stop},\sigma_{stop},\tau_{stop}, P(TF), SSD)\\ &&\;\;= \prod\limits^{R}_{r=1} \left\{P(TF) \times f_{go}(t_{r};\mu_{go},\sigma_{go},\tau_{go})\right. \\ &&\;\;\;\;\; +\! \left[\!1\,-\,P(TF)\!\right] \!\times\! \left[\!1\,-\,F_{stop}(t_{r};\!\mu_{stop},\!\sigma_{stop},\!\tau_{stop},\! SSD)\!\right]\\ && \;\;\;\;\; \left.\times f_{go}(t_{r};\mu_{go},\sigma_{go},\tau_{go}) \right\} \\ \end{array} $$

where *f*_*go*_(*t*;*μ*_*go*_, *σ*_*go*_, *τ*_*go*_) is the probability density function of the ex-Gaussian go RT distribution and *F*_*stop*_(*t*;*μ*_*stop*_, *σ*_*stop*_, *τ*_*stop*_, *S* *S* *D*) is distribution function of the ex-Gaussian SSRT distribution for a given SSD. Note that *P*(*T* *F*) is assumed to be independent of SSD.

Inhibitions result from stop-signal trials where the stop process was successfully triggered. Successful inhibitions are therefore produced with 1−*P*(*T* *F*) if the stop process has finished before the go process (i.e., go RT > *S* *S* *R* *T* + *S* *S* *D*). Formally, the likelihood (*L*_*I*_) of the *i* = 1,..., *I* successful inhibitions for a given SSD is:

2 $$\begin{array}{@{}rcl@{}} &&\L_{I}(\mu_{go}, \sigma_{go},\tau_{go},\mu_{stop},\sigma_{stop},\tau_{stop}, P(TF),SSD) \\ &&\;\;\; =\! \prod\limits^{I}_{i=1} \left[1\,-\,P(TF) \right]\! \times\! {\int}_{\!\!\!\!-\infty}^{\infty} \left[1-F_{go}(t_{i};\mu_{go},\sigma_{go},\tau_{go})\right]\\ &&\;\;\;\;\;\;\times f_{stop}(t_{i};\mu_{stop},\sigma_{stop},\tau_{stop},SSD) dt_{i}, \end{array} $$

where *F*_*go*_(*t*;*μ*_*go*_, *σ*_*go*_, *τ*_*go*_) is the distribution function of the ex-Gaussian go RT distribution and *f*_*stop*_(*t*;*μ*_*stop*_, *σ*_*stop*_, *τ*_*stop*_, *S* *S* *D*) is the probability density function of the ex-Gaussian SSRT distribution for a given SSD. As SSRTs are unobservable, computing the likelihood of successful inhibitions involves integrating over *t*. Note that the integral in Eq. 2 acts as a normalizing constant for the probability density function of the go RTs in the second term of Eq. 1, ensuring that the distribution of signal-respond RTs integrates to 1 (see also Colonius et al., 2001; Logan et al., 2014).

#### Bayesian parameter estimation

As a result of the Bayesian formulation, the trigger-failure approach may be applied to hierarchical as well as individual data structures. In the individual model, we estimate parameters for each participant separately by updating the prior distributions with the incoming data to arrive at the posterior distributions. The priors quantify existing knowledge about the parameters. As in Matzke et al. (2013b), the priors for the go (*μ*_*go*_, *σ*_*go*_, and *τ*_*go*_) and stop parameters (*μ*_*stop*_, *σ*_*stop*_, and *τ*_*stop*_) are weakly informative uniform distributions. We assume a non-informative uniform prior for *P*(*T* *F*) that covers the entire allowable range between 0 and 1. The resulting posteriors quantify the uncertainty about the parameters after the data have been observed. The 95 % credible interval of the posterior extends from the 2.5^*th*^ to the 97.5^*th*^ percentile of the distribution and encompasses the range of values that—with 95 % probability—contains the true value of the parameter. The central tendency of the posterior, such as the median, is often used as a point estimate for the parameter.

In the hierarchical model, rather than estimating parameters separately for each participant, we explicitly model the between-subject variability of the parameters with group-level distributions (e.g., Gelman and Hill, 2007; Rouder et al., 2005). The group-level distributions act as priors to adjust or “shrink” extreme estimates to more moderate values. The degree of shrinkage is determined by the relative uncertainty of the parameter estimates. Especially in data sets with relatively few observations per participant, hierarchical estimation can provide more accurate and less variable estimates than the individual approach (Farrell & Ludwig, 2008). Moreover, hierarchical modeling automatically provides inference on both the individual and group levels.

We assume that the individual go and stop parameters are drawn from truncated normal distributions. For instance, each participant’s *μ*_*stop*_ parameter comes from a normal group-level distribution truncated at 0 and 1000 ms, with mean $\mu _{\mu _{stop}}$ and standard deviation $\sigma _{\mu _{stop}}$. Note that the upper truncation is not necessary, but is numerically helpful. As in Matzke et al. (2013b), the priors for the group-level means and group-level standard deviations are weakly informative uniform distributions. The participant-level *P*(*T* *F*) parameters are first projected from the probability scale to the real line with a “probit” (i.e., standard normal cumulative distribution function) transformation (see also Matzke et al., 2015; Rouder et al., 2008). The probit-transformed *P*(*T* *F*) parameters are then modeled with a normal group-level distribution truncated at −6 and 6. The group-level mean for *P*(*T* *F*) is assigned a standard normal prior truncated at −6 and 6; the group-level standard deviation is assigned a weakly informative uniform prior.

We used Metropolis-within-Gibbs sampling (Tierney 1994) to approximate the posterior distribution of the model parameters. For all reported analyses, we ran multiple sampling sequences (i.e., chains) and computed the $\hat {R}$ (Gelman and Rubin 1992) statistic to ascertain that the chains converged to their stationary distribution ($\hat {R} <1.1$). The parameter estimation routine is implemented in the BEESTS software (Matzke et al. 2013a) and is available at http://dora.erbe-matzke.com/software.html.

### Parameter recovery studies

#### Asymptotic performance

**Fig. 3:**
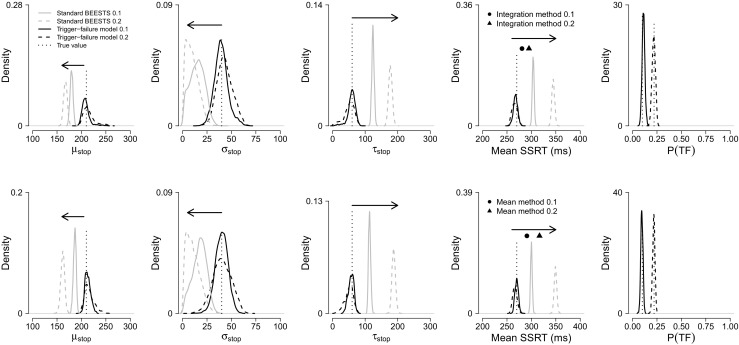
Asymptotic parameter recovery for the individual trigger-failure model, the standard BEESTS model, and the traditional integration and mean methods. The *top row* shows results for the fixed-SSD procedure. The *bottom row* shows results for the staircase tracking procedure. The *black posterior* distributions were computed with the trigger-failure model. The *gray posteriors* were computed with the standard BEESTS model. The posteriors plotted with *solid lines* are for the *P*(*T* *F*) = 0.1 scenario. The posteriors plotted with *dashed lines* are for the *P*(*T* *F*) = 0.2 scenario. The *vertical dotted lines* represent the true values, and *arrows* indicate the direction of the estimation bias. The *circle* and the *triangle* in the top row represent SSRTs computed with the integration method for *P*(*T* *F*) = 0.1 and *P*(*T* *F*) = 0.2, respectively. The *circle* and the *triangle* in the bottom row represent SSRTs computed with the mean method for *P*(*T* *F*) = 0.1 and *P*(*T* *F*) = 0.2, respectively

We first conducted a simulation study to examine asymptotic (i.e., large sample) SSRT estimation for a single participant with the trigger-failure model. We then investigated the bias in SSRT estimates caused by the presence of trigger failures in the standard BEESTS model and in the traditional integration and mean SSRT-estimation methods. We generated four stop-signal data sets from the race model using the ex-Gaussian distribution. The first and second data sets were generated using the fixed-SSD procedure with *P*(*T* *F*) = 0.1 and *P*(*T* *F*) = 0.2, respectively. In the first data set, overall signal-respond rate equaled 0.5; in the second data set, it equaled 0.57. The third and fourth data sets were generated using staircase tracking with *P*(*T* *F*) = 0.1 and *P*(*T* *F*) = 0.2, respectively. Each data set contained a total of 12,500 stop-signal trials. The generating parameter values are shown in Fig. 3. Note that we also assessed parameter recovery using a range of different true parameter values; the results were qualitatively similar to the ones reported here.

The Bayesian results reported below are based on 6,000 retained posterior samples per data set. Further technical details of the sampling run and information about the specification of the priors are presented in the Supplemental Material available at http://dora.erbe-matzke.com/publications.html. For the fixed-SSD data sets, we computed traditional SSRT estimates with the integration method, the most popular method for the fixed delays. For the staircase data sets, we computed traditional SSRT estimates with the mean method, which is most commonly used with staircase tracking (for overview of these methods, see Verbruggen et al. 2008, 2013).

#### Performance under realistic circumstances

**Fig. 4:**
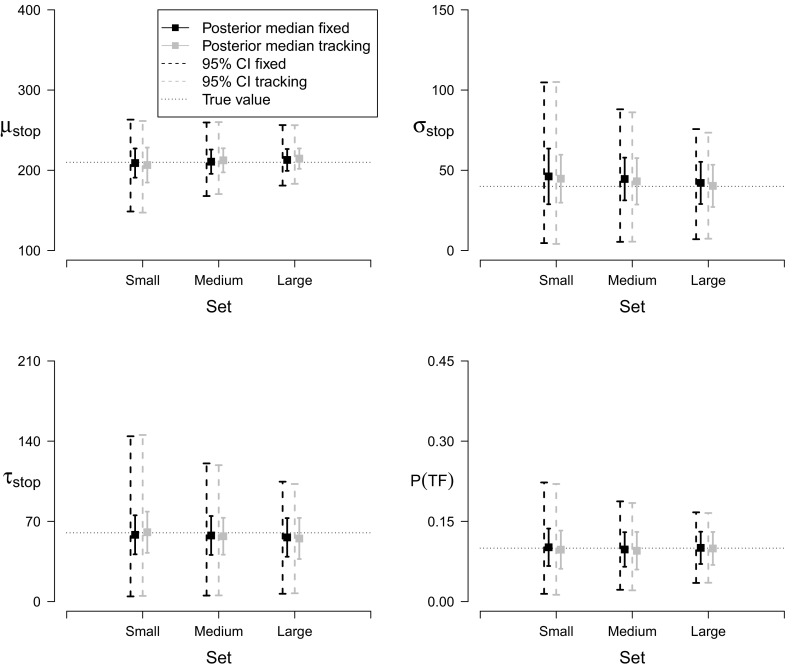
Parameter recovery for the individual trigger-failure model for small, medium, and large data sets. The *black* and *gray squares* show the average of the posterior medians across the 100 replications for the fixed-SSD and the staircase tracking procedure, respectively. The *error bars* represent the standard error of the posterior median. The *black* and *gray dashed lines* show the average range of the 95 % credible intervals across the 100 replications for the fixed-SSD and the staircase tracking procedure, respectively. The *dotted horizontal lines* represent the true values

We next conducted a series of simulations examining the effect of sample size on SSRT estimation by both the individual and the hierarchical trigger-failure models. For the individual case, we assessed parameter recovery for *P*(*T* *F*) = 0.1 and investigated the performance of both the fixed-SSD and the staircase tracking procedures. For both SSD methods, we manipulated the number of observations over three levels: 750 go trials and 250 stop-signal trials (small set); 1500 go trials and 500 stop-signal trials (medium set); and 3000 go trials and 1000 stop-signal trials (large set). For each 6=2 (SSD procedure) × 3 (Number of observations) scenario, we generated 100 stop-signal data sets from the race model using the ex-Gaussian distribution. The generating parameter values are shown in Fig. 4. Each data set was fit individually, with the results reported below based on 6,000 posterior samples retained per data set.

**Fig. 5:**
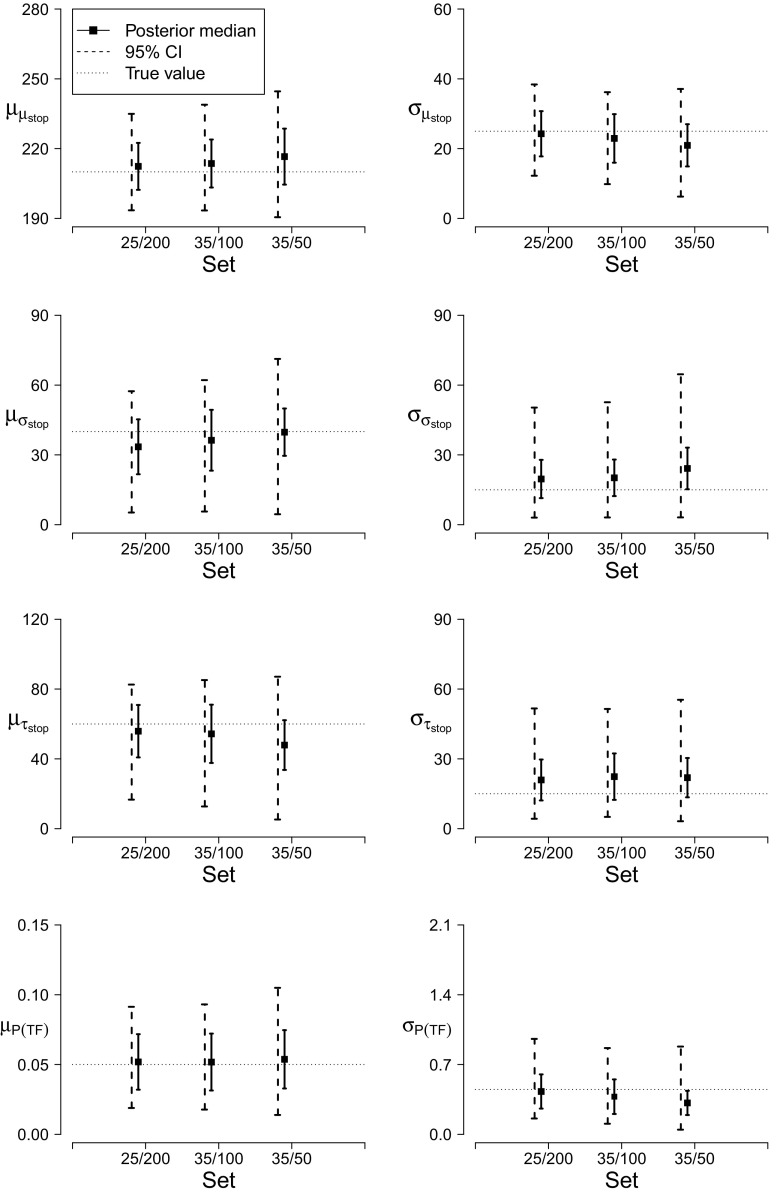
Parameter recovery for the hierarchical trigger-failure model for three sample sizes. The *black squares* show the average of the posterior medians across the 100 replications. The *error bars* represent the standard error of the posterior median. The *black dashed lines* show the average range of the 95 % credible intervals across the 100 replications. The *horizontal dotted lines* represent the true values. The group-level mean of *P*(*T* *F*) has been transformed back to the probability scale; the group-level standard deviation is on the probit scale. 25/200=25 participants and 200 stop-signal trials per participant; 35/100=35 participants and 100 stop-signal trials per participant; 35/50=35 participants and 50 stop-signal trials per participant

For the hierarchical case, we assessed parameter recovery only with staircase tracking because the majority of recent stop-signal studies have relied on it to set SSDs (Verbruggen et al. 2013). We manipulated the number of participants and the number of observations per participant over three levels: 25 participants, 600 go trials, and 200 stop-signal trials per participant (25/200 set); 30 participants, 300 go trials, and 100 stop-signal trials per participant (35/100 set); and 35 participants, 150 go trials, and 50 stop-signal trials per participant (35/50 set). We generated 100 stop-signal data sets per scenario as follows. For each data set, the participant-level go and stop parameters and the probit transformed *P*(*T* *F*) parameters were drawn from truncated normal distributions, with group-level means and standard deviations as shown in Fig. 5. The participant-level parameters were then used to generate stop-signal data for each synthetic participant using the ex-Gaussian distribution. Each of the resulting groups of 100 data sets was fit with the hierarchical trigger-failure model. The results reported below are based on 6,000 retained posterior samples per group.

### Illustrative published stop-signal data sets

Finally, we compared the performance of the hierarchical version of the trigger failure and the standard BEESTS models using stop-signal data from healthy controls in two studies of inhibition deficits in schizophrenia. The data set from Hughes et al. (2012) had 13 participants, each performing 672 go and 224 stop-signal trials. SSDs were set with the staircase tracking procedure. Following Hughes et al., we only used go RTs of correct responses, resulting in 3 % of the trials being excluded.

The data set from Badcock et al. (2002) had 30 participants, each performing eight blocks of 36 go and 12 stop-signal trials. In each block, SSDs were based on each participant’s go RT from the preceding block: stop signals were presented 500, 400, 300, 200, 100, and 0 ms prior to the last-block mean go RT. In each block, stop signals occurred twice at each of the six SSDs. We only used go RTs of correct responses and removed go RTs that were slower or faster than each participant’s mean go RT plus or minus three times the standard deviation, resulting in exclusion of 3 % of the trials. We also removed signal-respond RTs that were faster than 250 ms, excluding a further 1 % of the trials. For both data sets, the results reported below are based on 6,000 retained posterior samples. Further technical details of the sampling run and information about the prior setting are available in the Supplemental Material.

We assessed the relative goodness-of-fit of the trigger failure and the standard BEESTS models using the Deviance Information Criterion (DIC; Spiegelhalter et al., 2002), a popular model selection index that is particularly suited for comparing the performance of Bayesian hierarchical models. The model with the smaller DIC value is better supported by the data. A DIC difference of more than 10 can be considered strong evidence; a difference between 5 and 10 substantial evidence; and a difference of less than 5 only weak evidence for the model with the lower DIC value.

We assessed the absolute goodness-of-fit of the preferred model using posterior predictive model checks (Gelman et al. 1996). Posterior predictive checks evaluate the descriptive accuracy of a model by comparing predictions based on the posterior distribution of the model parameters to the observed data. If the model adequately describes the data, the predictions should closely approximate the observed data. As a result of using the entire posterior distribution to generate predictions, posterior predictive checks automatically take into account uncertainty about parameter estimates.

We preformed the posterior predictive model checks using the go RT distribution and the signal-respond rate of the individual participants. We randomly selected 1,000 parameter vectors from the joint posterior of the participant-level model parameters. We then generated 1,000 stop-signal data sets using the chosen parameter vectors. For the go RTs, we visually compared the observed go RT distribution to the 1,000 go RT distributions predicted by the model. For signal-respond rate, we visually compared the observed and predicted rates at each SSD, and quantified misfit with posterior predictive *p* values, which are computed for each SSD as the fraction of predicted signal-respond rates greater than the observed rate. On SSDs where signal-respond rate equaled 1, *p* values were computed as the fraction of time that the predicted signal-respond rate was greater than or equal to the observed signal-respond rate. Extreme *p* values indicate that the model does not provide an adequate description of the observed data.

## Results

### Parameter recovery studies

#### Asymptotic performance

Figure 3 shows the asymptotic performance of the different estimation methods in the presence of trigger failures. The top row shows the results for the fixed-SSD procedure; the bottom row shows the results for staircase tracking. The first three columns compare the recovery of the stop parameters estimated with the trigger failure and the standard BEESTS models. The black posteriors plotted with solid and dashed lines are computed with the trigger-failure model in the 10 and 20 % trigger failure scenarios, respectively. The gray posteriors plotted with solid and dashed lines are computed with the standard BEESTS model in the 10 and 20 % trigger failure scenarios, respectively. The vertical dotted lines represent the true values.

The trigger-failure model recovered the true value of the stop parameters very well regardless of the SSD procedure. The parameters were estimated precisely (i.e., peaked posteriors) and the true values were well within the 95 % credible interval of the posterior distributions. The estimates for the *P*(*T* *F*) = 0.1 scenario are generally more precise than the estimates for *P*(*T* *F*) = 0.2, consistent with the fact that the data sets with 10 % trigger failures contain more information about the stop parameters as the stop process is active on more trials. Importantly, as shown in the fifth column, recovery of *P*(*T* *F*)—the true probability of trigger failures—was excellent in all four data sets.

In contrast to the trigger-failure model, the standard BEETS model underestimated *μ*_*stop*_ and *σ*_*stop*_, and severely overestimated *τ*_*stop*_ for both SSD procedures. The bias and the uncertainty of the estimates increased with increasing *P*(*T* *F*). As shown in the fourth column of Fig. 3, the bias resulted in a dramatic overestimation of mean SSRT (i.e., *μ*_*stop*_ + *τ*_*stop*_). Traditional estimation methods also overestimated SSRT, although to a lesser degree. The bias increased with increasing *P*(*T* *F*) and was larger for the mean method in combination with staircase tracking (bottom panel) than for the integration method with fixed SSDs (top panel; see also Band et al., 2003).

#### Performance under realistic circumstances

Figure 4 shows recovery performance with the individual trigger-failure model for small, medium, and large data sets. We focus on the recovery of the *P*(*T* *F*) and the stop parameters; the results for the go parameters are available in the Supplemental Material. Regardless of the SSD procedure, the trigger-failure model recovered the generating parameter values adequately even with as few as 250 stop-signal trials (i.e., the small set). As the number of observations increased, the standard error of the posterior median and the range of the credible intervals decreased. The coverage of the 95 % credible intervals was satisfactory: depending on the number of observations per data set, the credible intervals contained the true values of the parameters in 96 to 100 % of the replications.

Figure 5 shows recovery performance with the hierarchical trigger-failure model. We focus on the recovery of the group-level stop and *P*(*T* *F*) parameters; the results for the group-level go parameters are available in the Supplemental Material. The trigger-failure model recovered the generating group-level parameters adequately for all sample sizes. For the group-level means, the standard error of the posterior median and the range of the credible intervals were the smallest for data sets with 25 participants and 200 stop-signal trials, increased slightly for data sets with 35 participants and 100 stop-signal trials, and were the largest for data sets with 35 participants and 50 stop-signal trials. For the group-level standard deviations, the standard errors and the credible intervals generally increased with the total number of observations, being the smallest for data sets with 25 participants and 200 stop-signal trials and the largest for data sets with 35 participants and 50 stop-signal trials. The coverage of the 95 % credible intervals was satisfactory: depending on sample size, the credible intervals contained the true values of the parameters in 92 to 100 % of the replications.

### Illustrative published stop-signal data sets

#### Hugher et al. (2012) data set

**Fig. 6:**
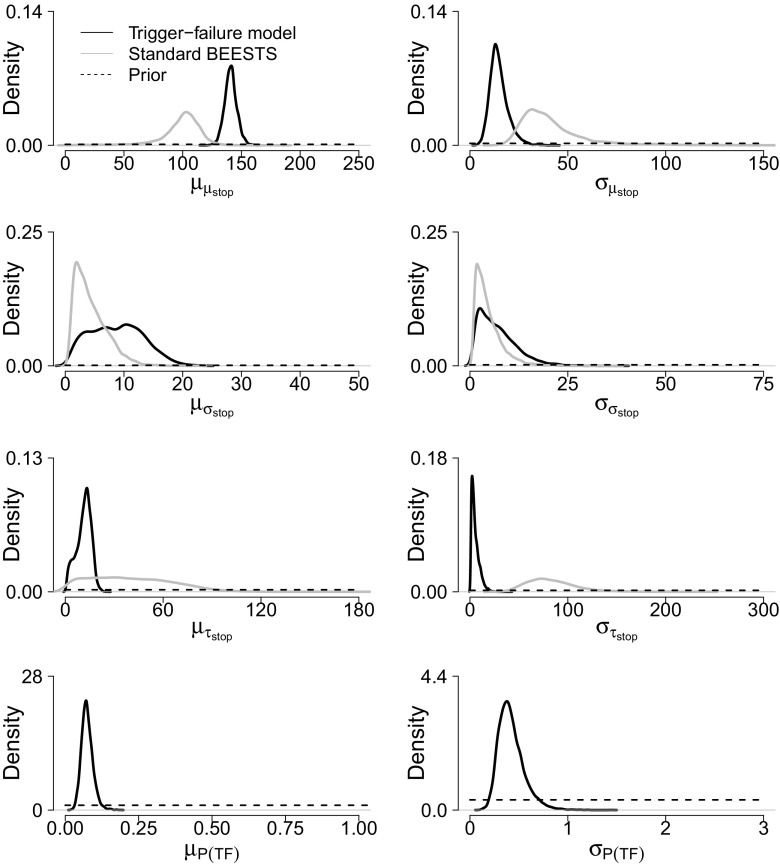
Group-level stop and *P*(*T* *F*) parameters for the Hughes et al. (2012) data set. The *first column* shows the group-level means. The *second column* shows the group-level standard deviations. The *black posteriors* are estimated with the trigger-failure model. The *gray* *posteriors* are estimated with the standard BEESTS model. The *dashed horizontal lines* represent the priors. The group-level mean of *P*(*T* *F*) is plotted on the probability scale; the group-level standard deviation is on the probit scale

Figure 6 shows the posterior distribution of the group-level means (first column) and group-level standard deviations (second column) of the stop and the *P*(*T* *F*) parameters. The results for the go parameters are available in the Supplemental Material. The black posteriors are estimated with the trigger-failure model; the gray posteriors are estimated with the standard BEESTS model. The group-level parameters for *μ*_*stop*_ and especially for *τ*_*stop*_ were estimated more precisely with the trigger-failure model than with the standard BEESTS model. The group-level means followed the pattern of the asymptotic recovery results with the individual model: compared to the trigger-failure analysis, the posteriors of $\mu _{\mu _{stop}}$ and $\mu _{\sigma _{stop}}$ were shifted to lower values, and the posterior of $\mu _{\tau _{stop}}$ was shifted to higher values in the standard BEESTS analysis. With respect to the group-level standard deviations, compared to the trigger-failure analysis, the posteriors of $\sigma _{\mu _{stop}}$ and $\sigma _{\tau _{stop}}$ were shifted to higher values in the standard BEESTS analysis. The group-level standard deviation $\sigma _{\sigma _{stop}}$ was roughly equal across the two modeling approaches.

As shown in the bottom row of Fig. 6, the different results can be explained by the presence of trigger failures. The posterior distribution of the group-level mean of *P*(*T* *F*) was shifted away from 0 with a median of 0.07, indicating the non-negligible presence of trigger failures. The DIC difference of 236 in favor of the trigger-failure model indicated that it (*D* *I* *C* = 114,643) provided a much better description of the data than the standard BEESTS model (*D* *I* *C* = 114,879).

To ascertain that the DIC has accurately recovered the true model that generated the data, we conducted a simulation study using the sample size and the parameter estimates from the Hughes et al. (2012) data set. In the first set of simulations, we generated 50 stop-signal data sets without trigger failures using the posterior mean of the group-level parameters obtained from fitting the standard BEESTS model. In the second set of simulations, we generated 50 stop-signal data sets with trigger failures using the posterior mean of the group-level parameters obtained from fitting the trigger-failure model. We then fit the 100 synthetic data sets both with the standard BEESTS and the trigger-failure models and assessed the difference in DIC.

When the data came from the standard BEESTS model, we were unable to compute the DIC difference between the models for 24 % of the data sets, because the chains for at least one of the parameters in the misspecified model—often the *P*(*T* *F*) parameter—failed to converge. For the remaining data sets, the DIC recovered the true model in 82 % of the cases, with an average DIC difference of 5.1. The average DIC difference for data sets with incorrect model recovery was 2.9. Both the standard BEESTS and the trigger-failure models recovered the generating values of the group-level stop parameters adequately, likely because the *P*(*T* *F*) parameters were estimated very close to 0. When the data came from the trigger-failure model, DIC recovered the true model in all of the data sets, with an average DIC difference of 110. The trigger-failure model recovered the generating values of the group-level *P*(*T* *F*) and stop parameters adequately, whereas the standard BEESTS analysis resulted in the same pattern of bias as for the observed data.

Our model recovery simulations found that when the data came from the standard BEESTS model, the stop estimates from the standard BEESTS and the trigger-failure model were similar, and the *P*(*T* *F*) parameter was estimated close to 0. When the data came from the trigger-failure model, the standard BEESTS analysis resulted in biased stop estimates, and the DIC indicated very strong preference for the trigger-failure model. These results corroborate our earlier conclusion that the trigger-failure model provided a better description of the Hughes et al. (2012) data than the standard BEESTS model: The standard BEESTS estimates were biased relative to the trigger-failure estimates, the posterior distribution of *P*(*T* *F*) was shifted away from 0, and the DIC evidence of 236 was clearly in favor of the trigger-failure model.

**Fig. 7:**
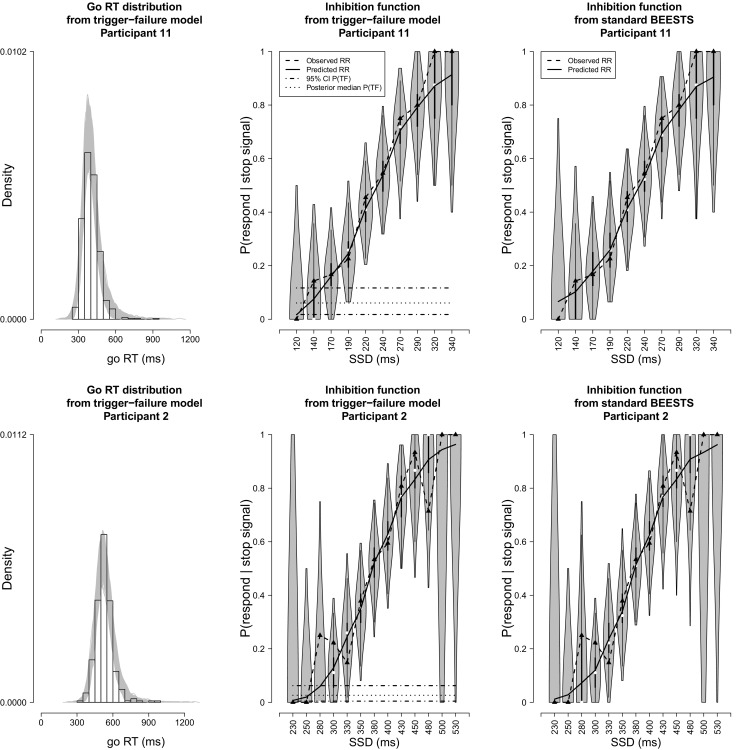
Posterior predictive model checks for two participants in the Hughes et al. (2012) data set. The *first column* shows histograms of the observed go RT distributions. The *gray lines* show 1,000 go RT distributions predicted by the trigger-failure model. The *dashed lines* in the second column show the observed signal-respond rates (RR) as a function of SSD. The *gray violin plots* show the distribution of 1,000 signal-respond rates predicted by the trigger-failure model on each SSD. The *black boxplot* in each violin plot ranges from the 25^*th*^ to the 75^*th*^ percentile of the predictions. The *black solid lines* connect the median of the predictions across the SSDs. The *dashed-dotted* and *dotted horizontal* lines show the 95 % credible interval (CI) and the median of the posterior distribution of the participant-specific *P*(*T* *F*) parameter, respectively. The *third column* shows the predicted signal-respond rates from the standard BEESTS analysis

Figure 7 shows the results of the posterior-predictive model checks for two participants using the joint posterior of the participant-level parameters from the preferred model, the trigger-failure model. The posterior-predictive checks for the remaining participants are available in the Supplemental Material. The first column shows histograms of the observed go RT distributions. The gray lines show the 1,000 predicted go RT distributions. For both participants, the predicted distributions closely followed the observed go RT distribution, indicating that the trigger-failure model provided a good description of the go RTs.

The dashed lines in the second column of Fig. 7 show the observed signal-respond rates as a function of SSD. As predicted by the race model, signal-respond rate increased with increasing SSD for both participants. The observed signal-respond rates were well within the range of signal-respond rates predicted by the trigger-failure model (i.e., gray violin plots), at least on the central SSDs where the tracking algorithm resulted in a reasonable number of stop-signal trials. This conclusion was corroborated by the posterior predictive *p* values: for SSDs with at least ten observed stop-signal trials, all *p* values were in an acceptable range (i.e., 0.05–0.95), indicating that the trigger-failure model provided a good description of the observed inhibition functions. Note that the participant-specific *P*(*T* *F*) parameters do not correspond to the asymptote of the empirical inhibition functions at short SSDs, likely because the staircase procedure sampled very few SSDs in this region.

For comparison, the third panel of Fig. 7 shows the results of the posterior predictive model checks for the standard BEESTS analysis. For both participants, the standard BEESTS model provided on average a reasonable description of the observed inhibition functions because the participant-specific *P*(*T* *F*) parameters were relatively low. For participant 11, however, the signal-respond rates predicted by the standard BEESTS model on early SSDs were clearly more variable than the ones predicted by the trigger-failure model. For participant 2, predictions from the two models were visually indistinguishable because the participant-specific *P*(*T* *F*) parameter was approaching 0.

#### Badcock et al. (2002) data set

**Fig. 8:**
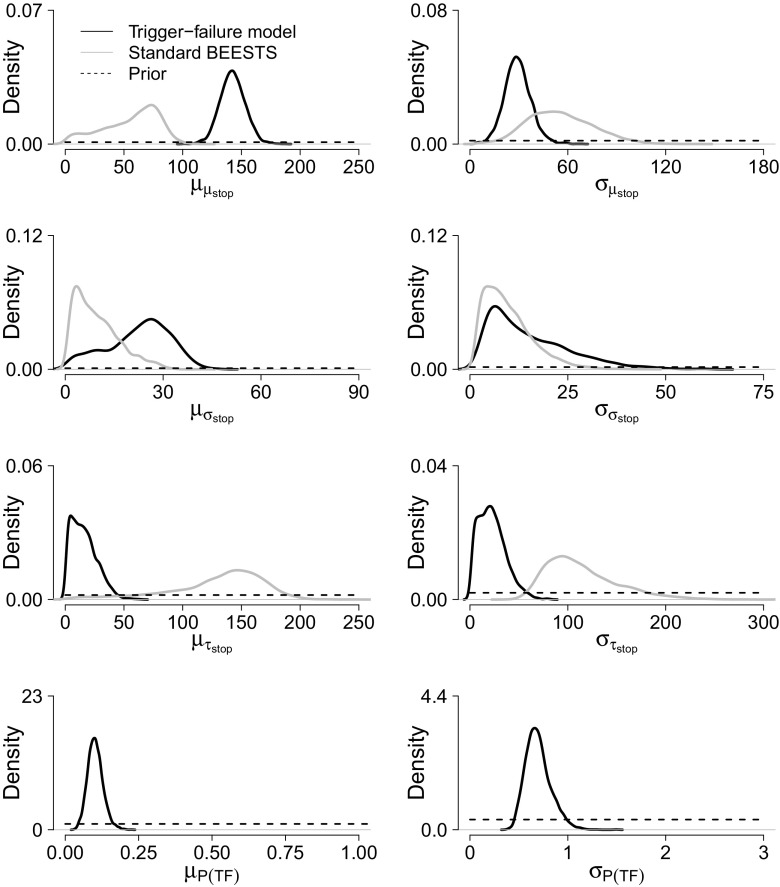
Group-level stop and *P*(*T* *F*) parameters for the Badcock et al. (2002) data set. See Fig. 6 for details

Figure 8 shows the posterior distribution of the group-level parameters. The results followed the same general pattern as for the Hughes et al. (2012) data. The posteriors estimated with the trigger-failure model were typically more precise than the posteriors estimated with the standard BEESTS model. Compared to the trigger-failure analysis, the posteriors of $\mu _{\mu _{stop}}$ and $\mu _{\sigma _{stop}}$ were shifted to lower values, and the posterior of $\mu _{\tau _{stop}}$ was shifted to higher values in the standard BEESTS analysis. The posteriors of $\sigma _{\mu _{stop}}$ and $\sigma _{\tau _{stop}}$ were shifted to higher values in the standard BEESTS analysis, and $\sigma _{\sigma _{stop}}$ was roughly equal across the two modeling approaches. Importantly, the group-level mean of *P*(*T* *F*) suggested the non-negligible presence of trigger failures, with a posterior median of 0.10. The DIC difference of 117 in favor of the trigger-failure model indicated that it (*D* *I* *C* = 116,693) provided a much better description of the data than the standard BEESTS model (*D* *I* *C* = 116,810).

**Fig. 9:**
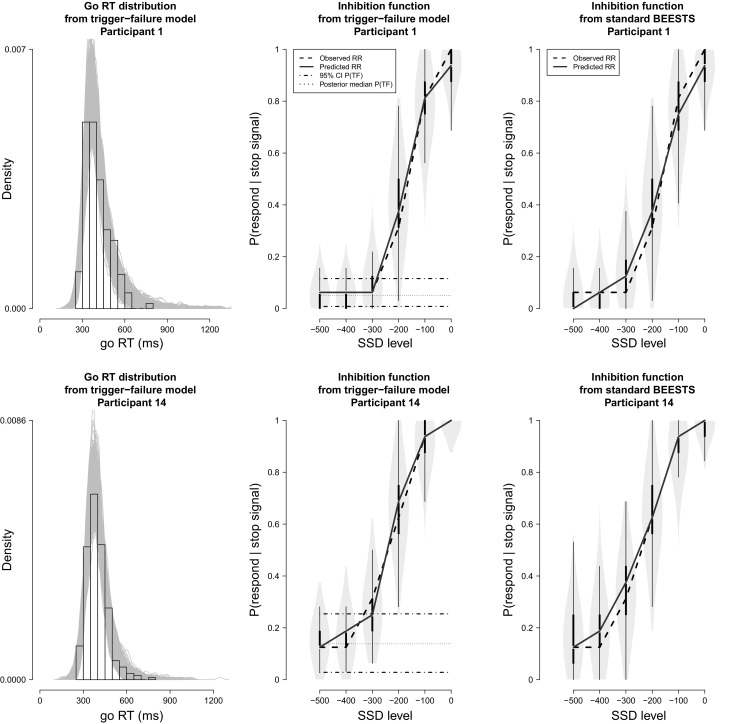
Posterior predictive model checks for two participants in the Badcock et al. (2002) data set. The *first* and *second panels* show the results of the posterior predictive model checks for the trigger-failure model; the *third panel* shows the results for the standard BEESTS analysis. See Fig. 7 for details

Figure 9 shows the results of the posterior predictive model checks for two participants. For both participants, the predicted go RT distributions closely followed the observed go RT distribution, indicating that the trigger-failure model provided a good description of the go RTs. The predicted signal-respond rates adequately approximated the observed signal-respond rates for all SSDs, and all *p* values were in an acceptable range, indicating adequate fit to the observed inhibition functions.

For both participants, the observed inhibition function asymptotes at short SSDs, where there are a reasonable number of stop-signal trials, and so should give a reasonable estimate of participants’ trigger failure probability. The second column of Fig. 9 shows the 95 % credible interval (dashed-dotted line) and the median (dotted line) of the posterior of the participant-specific *P*(*T* *F*) parameters. Indeed, the posterior median of the estimated *P*(*T* *F*) parameters very closely approximated the lower asymptote of the observed inhibition functions. About one-third of the participants had similar results, but for the remainder there was no clear asymptote in the inhibition function, even when *P*(*T* *F*) was estimated as clearly greater than zero. The Supplemental Material presents the full set of posterior predictive model checks as well as figures depicting the relationship between the participant-specific *P*(*T* *F*) parameters and the lower asymptote of the inhibition functions.

The third panel of Fig. 9 shows the results of the posterior predictive model checks for the standard BEESTS analysis. For Participant 1, the standard BEESTS model clearly failed to account for the lower asymptote of the observed inhibition function. Moreover, the signal-respond rates predicted by the standard BEESTS model were more variable than the ones predicted by the trigger-failure model, especially on early SSDs. For Participant 14, the standard BEESTS analysis seemed to account less well than the trigger-failure model for the observed signal-respond rates on SSD levels −400 and −300. Again, the predicted signal-respond rates on early SSDs were more variable that the ones generated by the trigger-failure model.

## Discussion

We introduced a Bayesian mixture model that allows researchers to estimate the probability of trigger failures as well as the entire distribution of stopping latencies in the stop-signal paradigm. We first showed that—in contrast to other methods—the trigger-failure approach accurately recovers SSRTs even in the presence of relatively frequent trigger failures. We then demonstrated that trigger failures play an important role in stop-signal performance even in participants chosen as healthy controls in two studies of inhibition deficits in schizophrenia. Although the level of trigger failure was relatively modest—around 8–9 % on average—its presence was shown to dramatically distort estimates of SSRTs.

Our approach to trigger failures was developed within a Bayesian framework, but parameter estimation in the individual model might also proceed with standard maximum likelihood estimation (Myung 2003). For the hierarchical model, however, maximum likelihood estimation can become computationally infeasible. Moreover, the present formulation allows us to take advantage of the benefits of Bayesian inference, such as a coherent inferential framework, the principled use of prior information, and the possibility of state-of-the-art model selection techniques. In either case, our approach does not depend on the particular ex-Gauss form that we use to describe the data (see also Matzke et al., 2013a, 2013b). The ex-Gauss may be substituted with other RT distributions, such as the Wald or Lognormal distributions (e.g., Heathcote, 2004; Heathcote et al., 2004). Our mixture-model based approach may be also used to augment recently developed process models of response inhibition (Logan et al. 2014).

We used weakly informative priors for parameter estimation and relied on the DIC to compare the goodness-of-fit of the trigger failure and standard BEESTS models. Note that Bayesian parameter estimation is robust to changes in the prior as long as sufficiently informative data are available (Lee and Wagenmakers 2013). As opposed to estimation, Bayesian model selection can be sensitive to the prior distributions. Our choice for the DIC was partly motivated by the fact that it does not require the explicit specification of prior information. Due to its strong dependence on the prior, the development of more sophisticated model selection measures, such as the Bayes factor (Berger 2006; Liu and Aitkin 2008), requires further research focusing on the choice of theoretically justified and computationally convenient prior distributions.

Our parameter recovery studies indicated that the trigger-failure model can provide accurate and precise parameter estimates with a realistic number of observations. Our findings with respect to the required number of observations, however, only serve as a rough rule of thumb. Recovery performance depends on the true—unknown—probability of trigger failures in a particular data set: the more prevalent trigger failures are, the less information the stop-signal trials provide about the stop parameters, and the more data are needed to obtain the same level of estimation precision. Prior to data collection, we encourage users to examine the expected uncertainty of the estimates with synthetic data by varying the probability of trigger failures and/or the number of observations per participant. Data from pilot participants can help guide choices about other parameters in these simulations.

The parameter recoveries have also shown that for signal-respond rate of approximately 0.50 recovery performance was similar for the two SSD procedures. However, the two procedures may yield results that differ in the relative precision of the parameter estimates. With the fixed-SSD procedure, it is possible to present participants with a large number of early SSDs that provide the most valuable information about the *P*(*T* *F*) parameter. However, the fixed-SSD procedure can also result in low overall signal-respond rate, which can hinder the estimation of the stop parameters. The tracking procedure results in relatively few stop-signal trials at early SSDs, but unless the probability of trigger failures approaches 50 %, it typically yields a sufficient number of signal-respond RTs to estimate the stop parameters. In practice, a hybrid SSD procedure might work ideally, with a proportion of stop-signal trials at early fixed SSDs to facilitate the estimation of *P*(*T* *F*), and the tracking algorithm on the remaining trials to obtain a sufficient number of signal-respond RTs for the precise estimation of the stop parameters.

Our model recovery simulations found that when the data came from the trigger-failure model, DIC-based model selection was correct and clear (i.e., provided very strong evidence). The situation was less straightforward for data sets without trigger failures. DIC-based selection identified the correct model in the majority of the data sets, but the DIC evidence for the standard model was only moderate. Therefore, we urge readers to visually compare the parameter estimates from the two models and to not only consider the DIC difference. If the DIC prefers the standard model, parameter estimates from the two models are similar, and the *P*(*T* *F*) parameters are estimated close to 0, it is safe to conclude that the standard model provides a better description of the data, even if the DIC evidence is not strong. However, in situations where the DIC difference is only slightly in favor of the trigger-failure model, parameter estimates from the two models are similar, and the *P*(*T* *F*) parameters are close to 0 (or have failed to converge), we discourage readers from interpreting the weak DIC evidence as sufficient support for the trigger-failure model.

## Conclusion

The goal of this paper was to introduce a Bayesian mixture model that enables researchers to investigate the relative contribution of trigger failures to stop-signal performance and to correct SSRT estimates for the bias that results from deficiencies in triggering the stop process. We illustrated the theoretical and methodological advantages of the proposed framework and demonstrated the clear presence of trigger failures in healthy populations. It seems likely that trigger failures will be at least as common, if not more so, in special populations, or under manipulations that tax participants’ cognitive resources. The way in which the probability of trigger failures varies with such factors will provide important theoretical and practical insights.

## Electronic supplementary material

Below is the link to the electronic supplementary material.

(PDF 117 MB)
