# Supplementary material for: A Bayesian approach for estimating the probability of trigger failures in the stop-signal paradigm
Source: Behav Res Methods. 2016 Jan 28;49(1):267–81. doi: 10.3758/s13428-015-0695-8 (PMC5352806; doi:10.3758/s13428-015-0695-8)
Supplement: Supplementary file 1 — (PDF 117 MB) [file 13428_2015_695_MOESM1_ESM.pdf]

# Supplemental Materials for A Bayesian Approach for Estimating the Probability of Trigger Failures in the Stop-Signal Paradigm

Dora Matzke[1], Jonathon Love[1], and Andrew Heathcote[2]
[1] University of Amsterdam, The Netherlands
[2]Universities of Tasmania and Newcastle, Australia

Correspondence concerning this article should be addressed to:
Dora Matzke
University of Amsterdam, Department of Psychology
Weesperplein 4
1018 XA, Amsterdam, The Netherlands
Phone: +31205258862
E-mail to d.matzke@uva.nl.

# Parameter Recover Studies

*Technical Details and Prior Settings for Recovery Studies with the Individual Trigger-Failure Model*

For further information, see the `Help` menu in the `BEESTS` software package available at `http://dora.erbe-matzke.com/software.html`.

```
"samples","25000"
"burn-in","5000"
"number of chains","3"
"thinning","10"
"estimates for subjects or groups","All"
"summary statistics","1"
"posterior distributions","1"
"mcmc chains","1"
"deviance","0"
"posterior predictors","0"
"posterior predictor samples","100"
"cpu cores","3"
"limits of integration lower","0"
"limits of integration upper","10000"
"model trigger failure","1"
"go mu lower","0.001"
"go mu upper","1000"
"go mu start","500"
"stop mu lower","0.001"
"stop mu upper","1000"
"stop mu start","300"
"go mu sd lower","1"
"go mu sd upper","500"
"go mu sd start","50"
"stop mu sd lower","1"
"stop mu sd upper","500"
"stop mu sd start","50"
"go sigma lower","1"
"go sigma upper","500"
"go sigma start","150"
"stop sigma lower","1"
"stop sigma upper","500"
"stop sigma start","150"
"go sigma sd lower","1"
"go sigma sd upper","500"
"go sigma sd start","50"
"stop sigma sd lower","1"
"stop sigma sd upper","500"
"stop sigma sd start","50"
"go tau lower","1"
"go tau upper","500"
"go tau start","150"
"stop tau lower","1"
"stop tau upper","500"
"stop tau start","150"
"go tau sd lower","1"
"go tau sd upper","500"
```

```
"go tau sd start","50"
"stop tau sd lower","1"
"stop tau sd upper","500"
"stop tau sd start","50"
"stop pf mean","0"
"stop pf sd","1"
"stop pf lower","0"
"stop pf upper","1"
"stop pf start","0.05"
"stop pf sd lower","0.01"
"stop pf sd upper","3"
"stop pf sd start","0.1"
```

*Technical Details and Prior Settings for Recovery Studies with the Hierarchical Trigger-Failure Model*

```
"samples","25000"
"burn-in","5000"
"number of chains","3"
"thinning","10"
"estimates for subjects or groups","All"
"summary statistics","1"
"posterior distributions","1"
"mcmc chains","1"
"deviance","0"
"posterior predictors","0"
"posterior predictor samples","100"
"cpu cores","3"
"limits of integration lower","0"
"limits of integration upper","10000"
"model trigger failure","1"
"go mu lower","0.001"
"go mu upper","1000"
"go mu start","500"
"stop mu lower","0.001"
"stop mu upper","1000"
"stop mu start","300"
"go mu sd lower","1"
"go mu sd upper","500"
"go mu sd start","50"
"stop mu sd lower","1"
"stop mu sd upper","500"
"stop mu sd start","50"
"go sigma lower","1"
"go sigma upper","500"
"go sigma start","150"
"stop sigma lower","1"
"stop sigma upper","500"
"stop sigma start","150"
"go sigma sd lower","1"
"go sigma sd upper","500"
"go sigma sd start","50"
"stop sigma sd lower","1"
"stop sigma sd upper","500"
"stop sigma sd start","50"
"go tau lower","1"
```

```
"go tau upper","500"
"go tau start","150"
"stop tau lower","1"
"stop tau upper","500"
"stop tau start","150"
"go tau sd lower","1"
"go tau sd upper","500"
"go tau sd start","50"
"stop tau sd lower","1"
"stop tau sd upper","500"
"stop tau sd start","50"
"stop pf mean","0"
"stop pf sd","1"
"stop pf lower","-6"
"stop pf upper","6"
"stop pf start","0.05"
"stop pf sd lower","0.01"
"stop pf sd upper","3"
"stop pf sd start","0.1"
```

*Parameter Recovery for the Go Parameters*

Figure 1 and Figure 2 show the recovery for the go parameters with the individual and the hierarchical trigger-failure models, respectively.

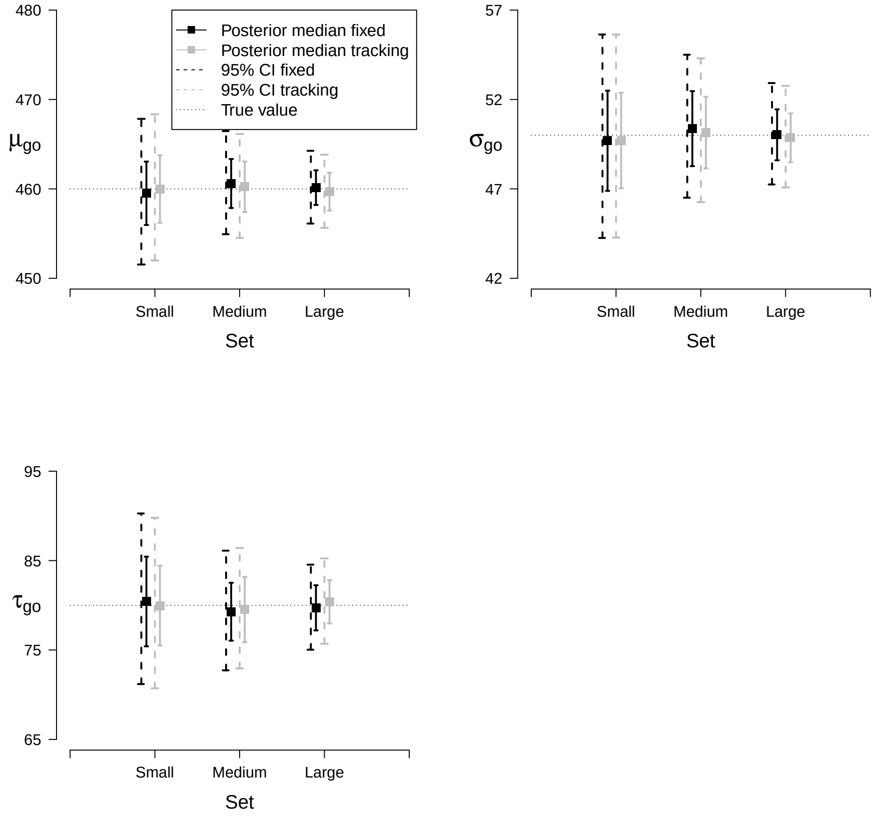


*Figure 1.* *Parameter recovery for the go parameters with the individual trigger-failure model for small, medium, and large data sets.* For the details of the simulations, the reader is referred to the main text. The black and gray squares show the average of the posterior medians across the 100 replications for the fixed-SSD and the staircase tracking procedure, respectively. The error bars represent the standard error of the posterior median. The black and gray dashed lines show the average range of the 95% credible intervals across the 100 replications for the fixed-SSD and the staircase tracking procedure, respectively. The dotted horizontal lines represent the true values.

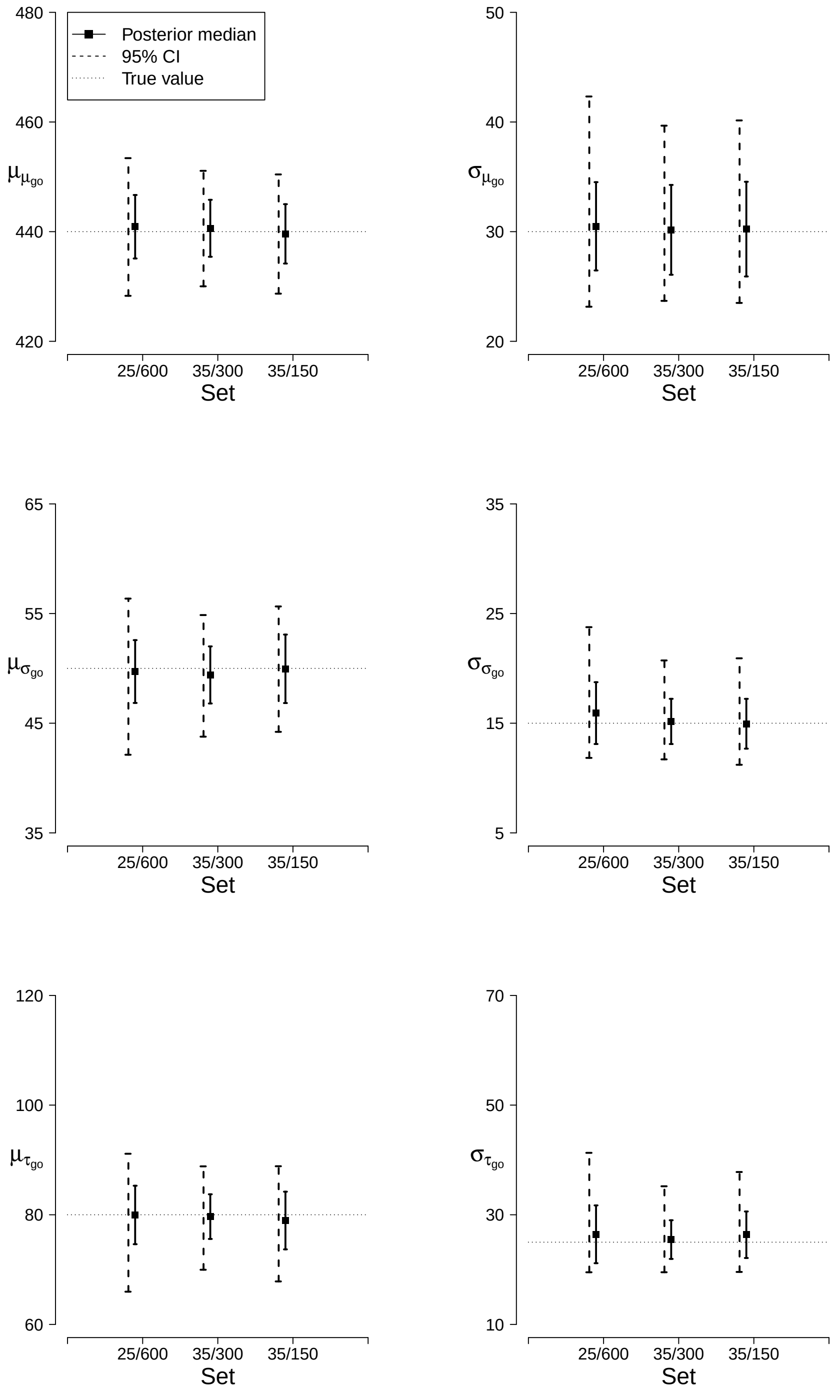


*Figure 2.* *Parameter recovery for the go parameters with the hierarchical trigger-failure model for three sample sizes.* For the details of the simulations, the reader is referred to the main text. The black squares show the average of the posterior medians across the 100 replications. The error bars represent the standard error of the posterior median. The black dashed lines show the average range of the 95% credible intervals across the 100 replications. The horizontal dotted lines represent the true values. 25/600 = 25 participants and 600 go trials per participant; 35/300 = 35 participants and 300 go trials per participant; 35/150 = 35 participants and 150 go trials per participant.

## Illustrative Published Stop-Signal Data Sets

*Technical Details and Prior Settings for the Analysis of the Hughes, Fulham, Johnston, and Michie (2012) and Badcock, Michie, Johnson, and Combrinck (2002) Data Sets with the Hierarchical Trigger-Failure Model*

```
"samples","25000"
"burn-in","5000"
"number of chains","3"
"thinning","10"
"estimates for subjects or groups","All"
"summary statistics","1"
"posterior distributions","1"
"mcmc chains","1"
"deviance","0"
"posterior predictors","0"
"posterior predictor samples","100"
"cpu cores","3"
"limits of integration lower","0"
"limits of integration upper","10000"
"model trigger failure","1"
"go mu lower","0.001"
"go mu upper","1000"
"go mu start","500"
"stop mu lower","0.001"
"stop mu upper","1000"
"stop mu start","300"
"go mu sd lower","1"
"go mu sd upper","500"
"go mu sd start","50"
"stop mu sd lower","1"
"stop mu sd upper","500"
"stop mu sd start","50"
"go sigma lower","1"
"go sigma upper","500"
"go sigma start","150"
"stop sigma lower","1"
"stop sigma upper","500"
"stop sigma start","150"
"go sigma sd lower","1"
"go sigma sd upper","500"
"go sigma sd start","50"
"stop sigma sd lower","1"
"stop sigma sd upper","500"
"stop sigma sd start","50"
"go tau lower","1"
"go tau upper","500"
"go tau start","150"
"stop tau lower","1"
"stop tau upper","500"
"stop tau start","150"
"go tau sd lower","1"
"go tau sd upper","500"
"go tau sd start","50"
"stop tau sd lower","1"
```

```
"stop tau sd upper","500"
"stop tau sd start","50"
"stop pf mean","0"
"stop pf sd","1"
"stop pf lower","-6"
"stop pf upper","6"
"stop pf start","0.05"
"stop pf sd lower","0.01"
"stop pf sd upper","3"
"stop pf sd start","0.1"
```

*Go Parameters and Posterior Predictive Model Checks for the Hughes et al. (2012) Data Set*

Figure 3 shows the posterior distribution of the group-level go parameters for the Hughes et al. (2012) data. Figures 4 - 16 show the results of the posterior predictive model checks for the individual participants.

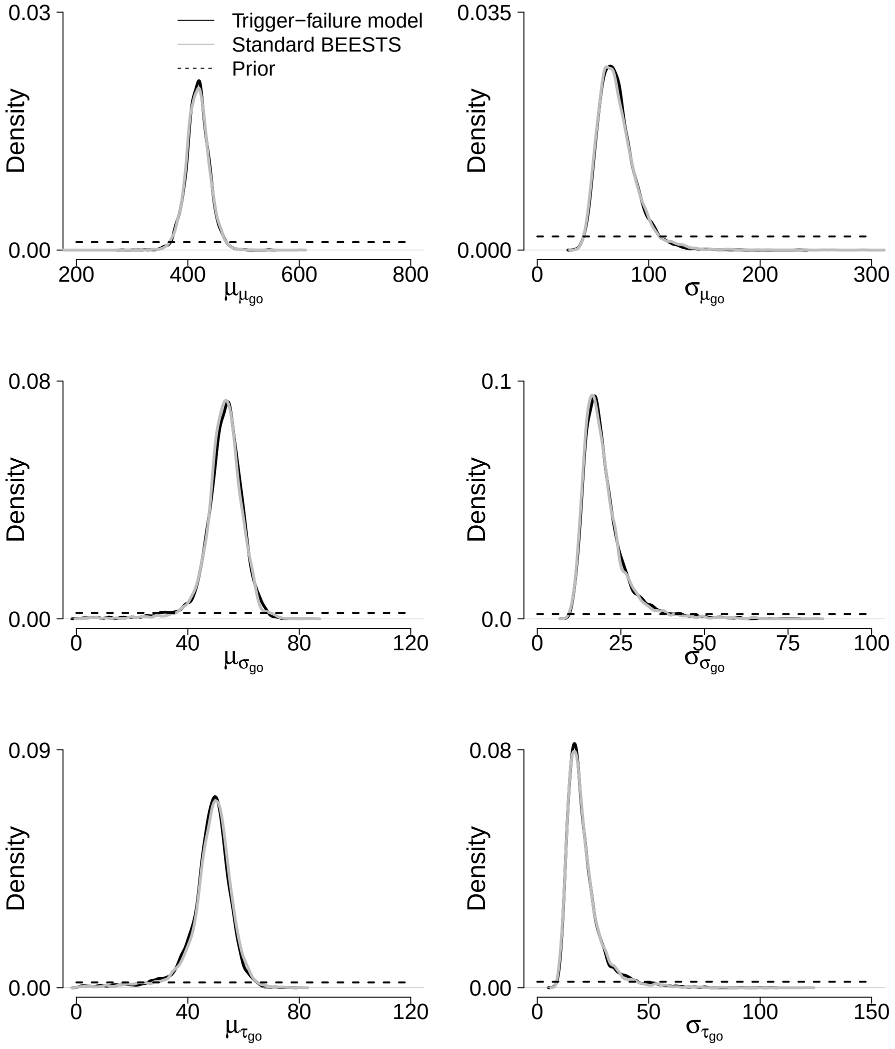


*Figure 3.* *Group-level go parameters for the Hughes et al. (2012) data set with the trigger failure and the standard BEESTS models.* The first column shows the group-level means. The second column shows the group-level standard deviations. The black posteriors are estimated with the trigger-failure model. The gray posteriors are estimated with the standard BEESTS model. The dashed horizontal lines represent the priors.

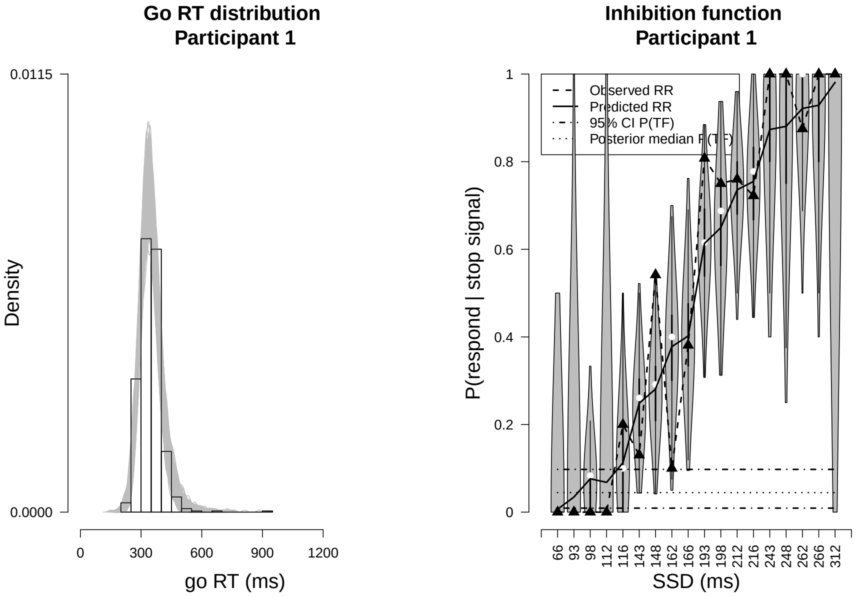


*Figure 4.* *Posterior predictive model checks for Participant* 1 *in the Hughes et al. (2012) data set.* For the details of the posterior predictive model checks, the reader is referred to the main text. The first column shows histograms of the observed go RT distributions. The gray lines show the 1,000 predicted go RT distributions. The dashed lines in the second column show the observed signal-respond rates (RR) as a function of SSD. The gray violin plots show the distribution of the 1,000 predicted response rates on each SSD. The black boxplot in each violin plot ranges from the $25^{th}$ to the $75^{th}$ percentile of the predictions. The black solid lines connect the median of the predictions across the SSDs. The dashed-dotted and dotted horizontal lines show the 95% credible interval and the median of the posterior distribution of the participant-specific $P(TF)$ parameter, respectively. The posterior predictive $p$ values on central SSDs with at least 10 observed stop-signal trials were 0.72 at 98 ms, 0.08 at 116 ms, 0.87 at 143 ms, 0.00 at 148 ms, 1.00 at 162 ms, 0.53 at 166 ms, 0.01 at 193 ms, 0.13 at 198 ms, 0.35 at 212 ms, and 0.57 at 216 ms.

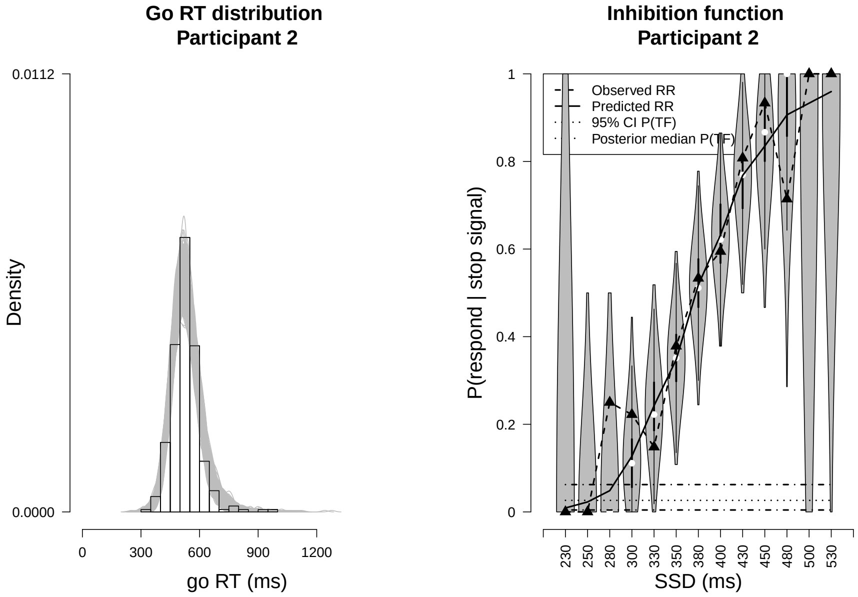


*Figure 5.* *Posterior predictive model checks for Participant* 2 *in the Hughes et al. (2012) data set.* The posterior predictive $p$ values on central SSDs with at least 10 observed stop-signal trials were 0.08 at 300 ms, 0.83 at 330 ms, 0.29 at 350 ms, 0.36 at 380 ms, 0.58 at 400 ms, 0.25 at 430 ms, and 0.07 at 450 ms.

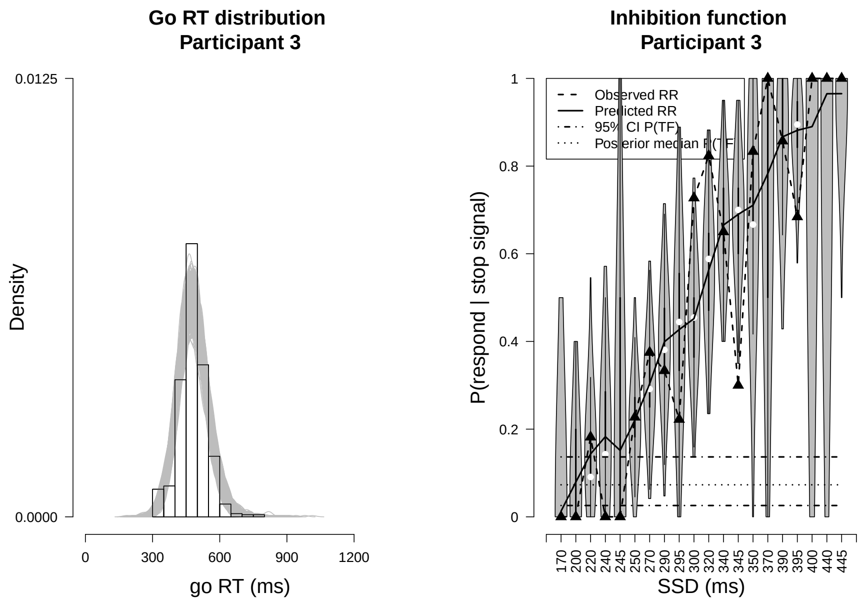


*Figure 6.* *Posterior predictive model checks for Participant* 3 *in the Hughes et al. (2012) data set.* The posterior predictive $p$ values on central SSDs with at least 10 observed stop-signal trials were 0.13 at 220 ms, 0.31 at 250 ms, 0.15 at 270 ms, 0.64 at 290 ms, 0.00 at 300 ms, 0.00 at 320 ms, 0.45 at 340 ms, 1.00 at 345 ms, and 0.98 at 395 ms.

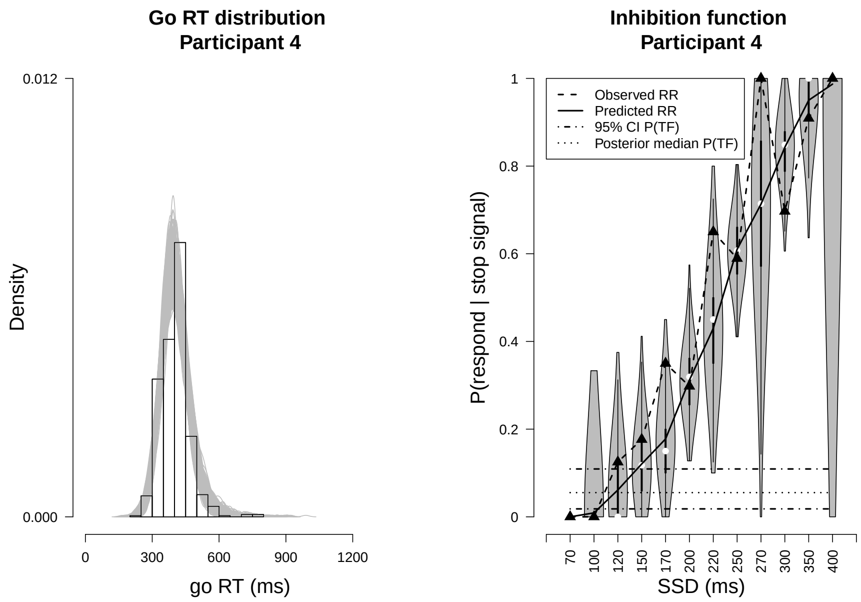


*Figure 7.* *Posterior predictive model checks for Participant* 4 *in the Hughes et al. (2012) data set.* The posterior predictive $p$ values on central SSDs with at least 10 observed stop-signal trials were 0.09 at 150 ms, 0.01 at 170 ms, 0.50 at 200 ms, 0.01 at 220 ms, 0.58 at 250 ms, 0.97 at 300 ms, and 0.56 at 350 ms.

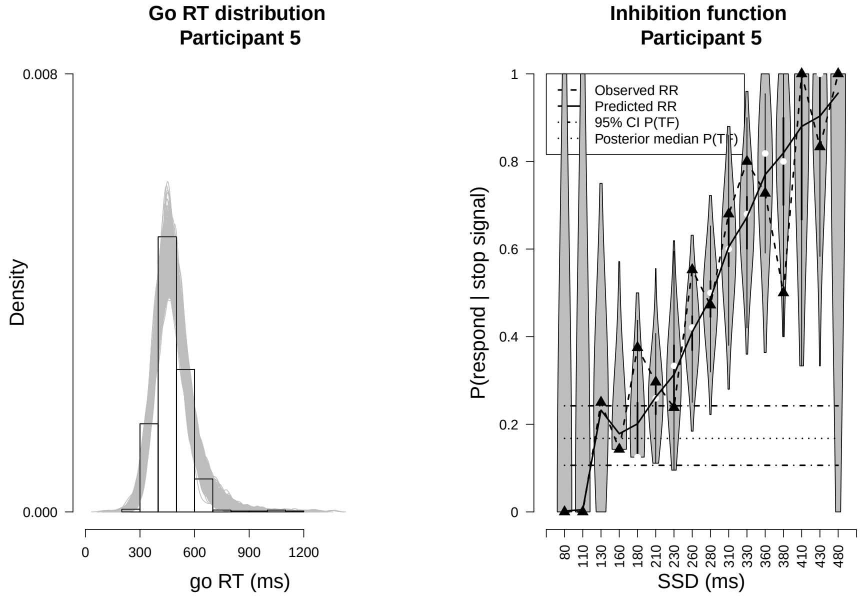


*Figure 8.* *Posterior predictive model checks for Participant* 5 *in the Hughes et al. (2012) data set.* The posterior predictive $p$ values on central SSDs with at least 10 observed stop-signal trials were 0.21 at 210 ms, 0.71 at 230 ms, 0.02 at 260 ms, 0.50 at 280 ms, 0.14 for 310 ms, 0.05 for 330 ms, 0.52 for 360, and 0.98 for 380 ms.

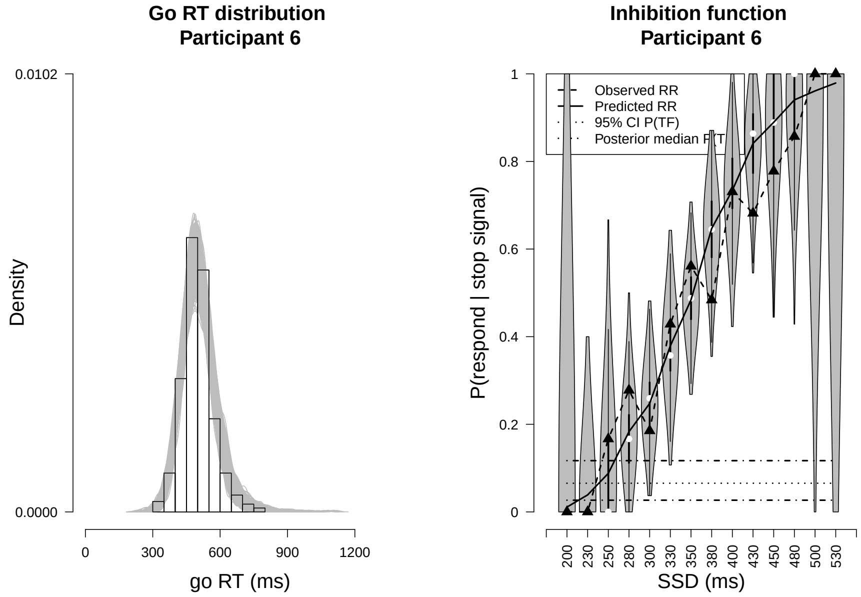


*Figure 9.* *Posterior predictive model checks for Participant* 6 *in the Hughes et al. (2012) data set.* The posterior predictive $p$ values on central SSDs with at least 10 observed stop-signal trials were 0.07 at 280 ms, 0.70 at 300 ms, 0.24 at 330 ms, and 0.15 at 350 ms, 0.94 for 380 ms, 0.46 for 400 ms, and 0.94 for 430.

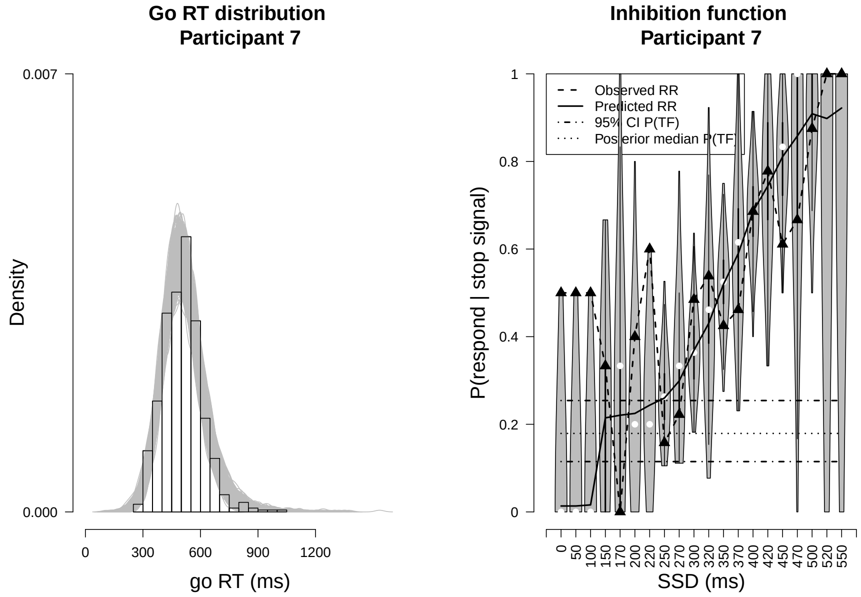


*Figure 10.* *Posterior predictive model checks for Participant* 7 *in the Hughes et al. (2012) data set.* The posterior predictive $p$ values on central SSDs with at least 10 observed stop-signal trials were 0.87 at 250 ms, 0.03 at 300 ms, 0.11 at 320 ms, 0.87 at 350 ms, 0.75 for 370 ms, 0.45 for 400 ms, and 0.97 for 450.

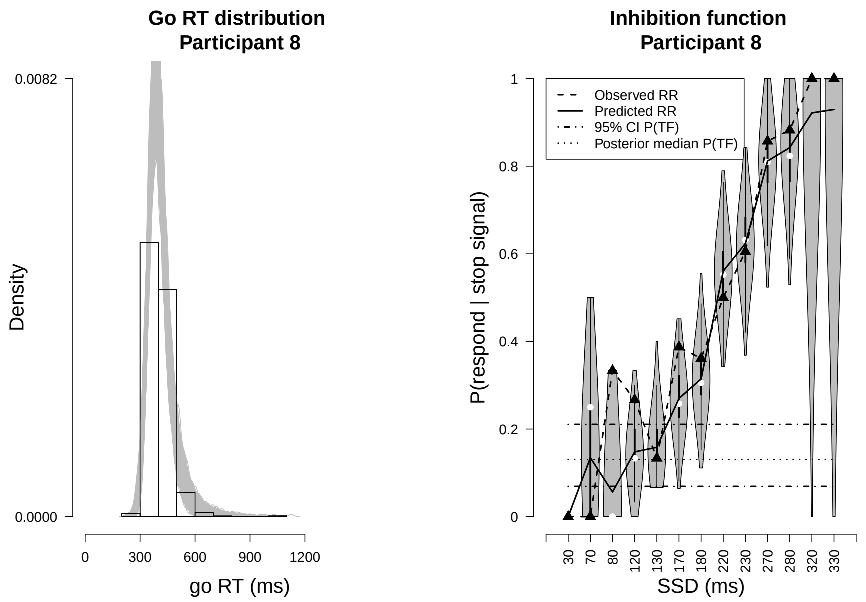


*Figure 11.* *Posterior predictive model checks for Participant* 8 *in the Hughes et al. (2012) data set.* The posterior predictive $p$ values on central SSDs with at least 10 observed stop-signal trials were 0.00 at 120 ms, 0.39 at 130 ms, 0.04 at 170 ms, 0.18 at 180 ms, 0.70 for 220 ms, 0.49 for 230 ms, 0.22 for 270, and 0.23 at 280 ms.

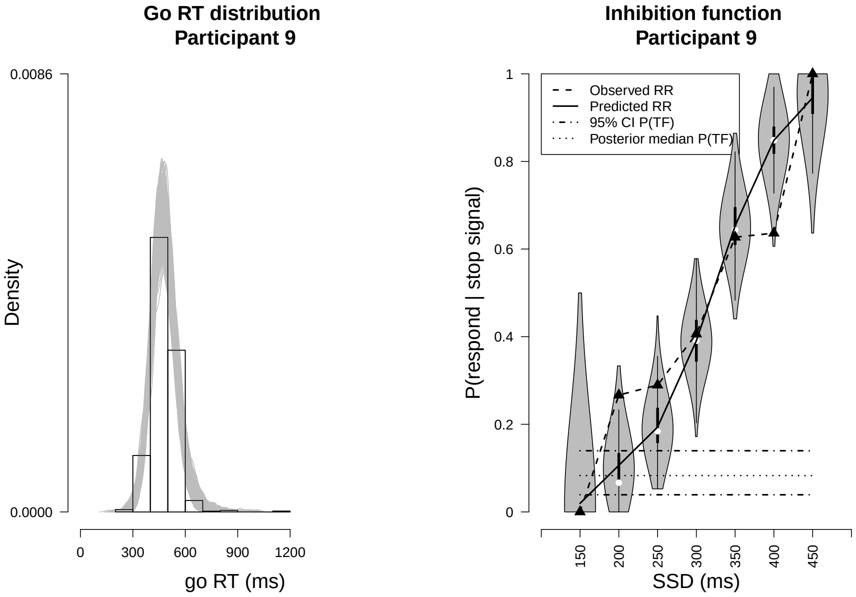


*Figure 12.* *Posterior predictive model checks for Participant* 9 *in the Hughes et al. (2012) data set.* The posterior predictive $p$ values on central SSDs with at least 10 observed stop-signal trials were 0.00 at 200 ms, 0.03 at 250 ms, 0.38 at 300 ms, 0.60 at 350 ms, 1.00 for 400 ms, and 0.55 for 450 ms.

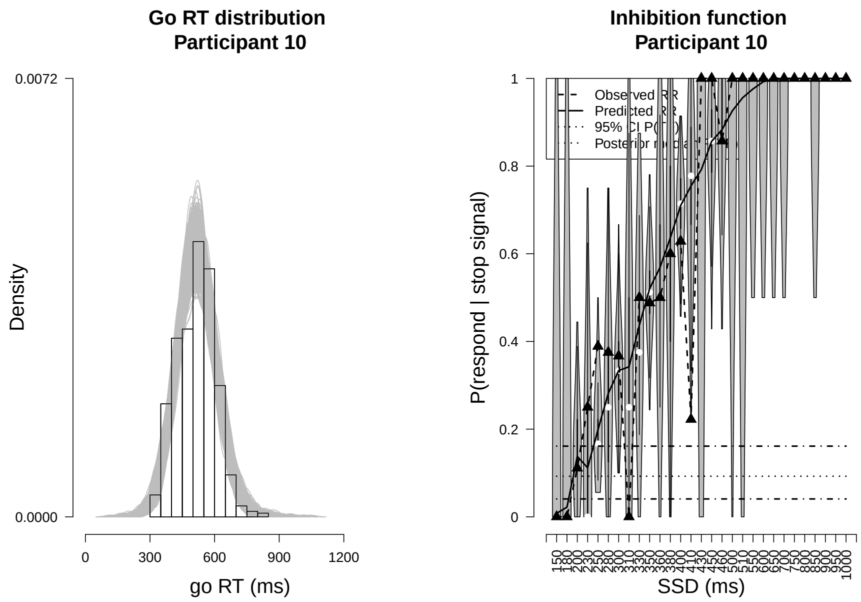


*Figure 13.* *Posterior predictive model checks for Participant* 10 *in the Hughes et al. (2012) data set.* The posterior predictive $p$ values on central SSDs with at least 10 observed stop-signal trials were 0.01 at 250 ms, 0.25 at 300 ms, 0.60 at 350 ms, 0.81 at 400 ms, and 0.13 for 450 ms.

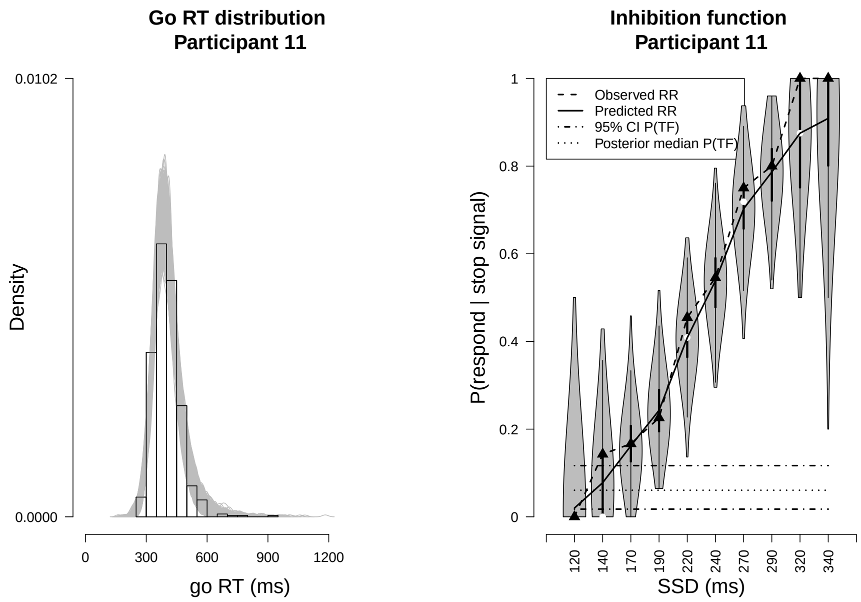


*Figure 14.* *Posterior predictive model checks for Participant* 11 *in the Hughes et al. (2012) data set.* The posterior predictive $p$ values on central SSDs with at least 10 observed stop-signal trials were 0.34 at 170 ms, 0.49 at 190 ms, 0.25 at 220 ms, 0.41 at 240 ms, 0.25 for 270 ms, and 0.39 for 290 ms.

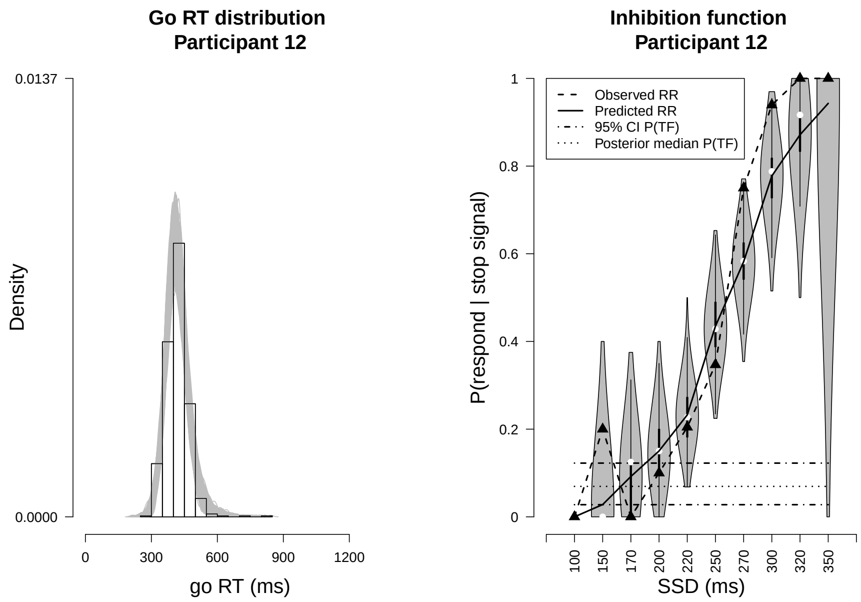


*Figure 15.* *Posterior predictive model checks for Participant* 12 *in the Hughes et al. (2012) data set.* The posterior predictive $p$ values on central SSDs with at least 10 observed stop-signal trials were 0.64 at 200 ms, 0.61 at 220 ms, 0.86 at 250 ms, 0.01 at 270 ms, 0.00 for 300 ms, and 0.19 for 320 ms.

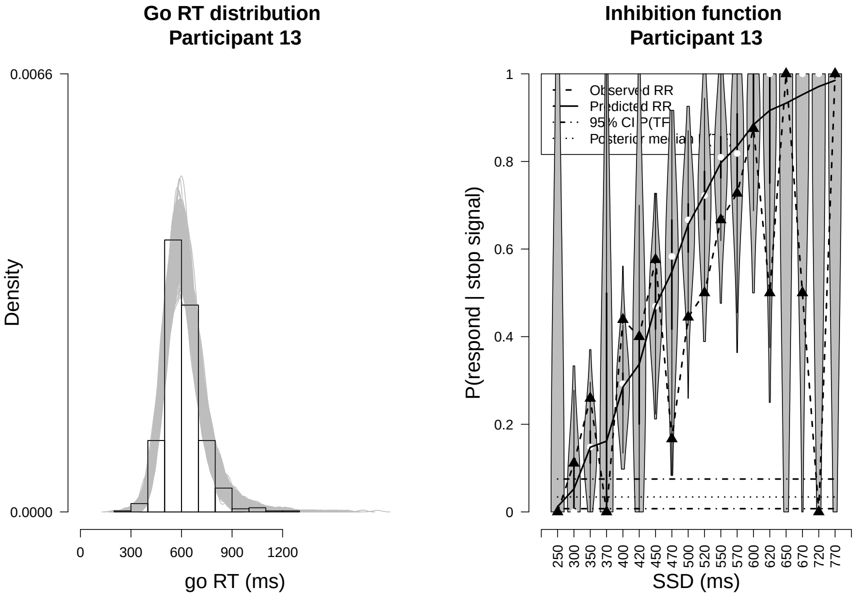


*Figure 16.* *Posterior predictive model checks for Participant* 13 *in the Hughes et al. (2012) data set.* The posterior predictive $p$ values on central SSDs with at least 10 observed stop-signal trials were 0.03 at 350 ms, 0.02 at 400 ms, 0.09 at 450 ms, 0.99 at 470 ms, 0.98 for 500 ms, 0.95 for 520 ms, 0.88 for 550 ms, and 0.75 for 570 ms.

### *Go Parameters and Posterior Predictive Model Checks for the Badcock et al. (2002) Data Set*

Figure 17 shows the posterior distribution of the group-level go parameters for the Badcock et al. (2002) data. Figures 18 - 47 show the results of the posterior predictive model checks for the individual participants. Figure 48 shows the relationship between the participant-specific $P(TF)$ parameters and observed signal-respond rate at the shortest SSD.

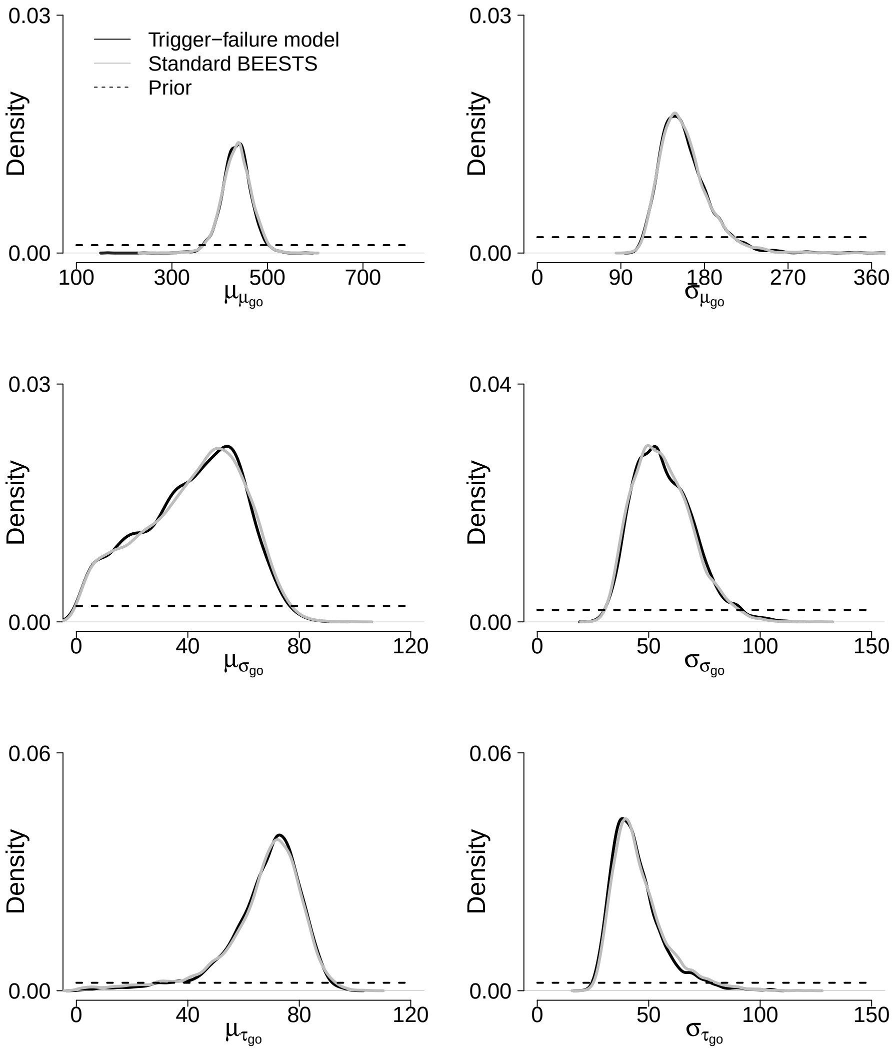


*Figure 17.* *Group-level go parameters for the Badcock et al. (2002) data set with the trigger failure and the standard BEESTS models.* The first column shows the group-level means. The second column shows the group-level standard deviations. The black posteriors are estimated with the trigger-failure model. The gray posteriors are estimated with the standard BEESTS model. The dashed horizontal lines represent the priors.

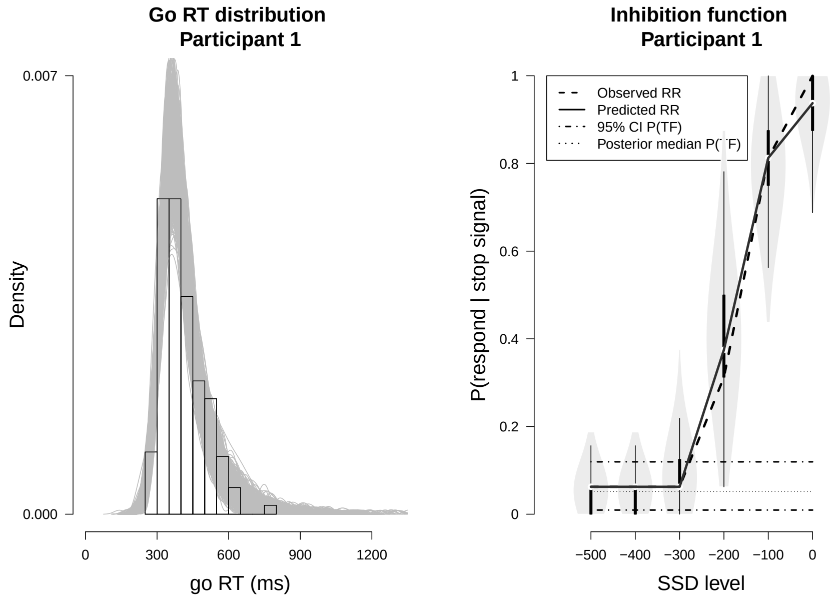


*Figure 18.* *Posterior predictive model checks for Participants* 1 *in the Badcock et al. (2002) data set.* For the details of the posterior predictive model checks, the reader is referred to the main text. The first column shows histograms of the observed go RT distributions. The gray lines show the $1,000$ predicted go RT distributions. The dashed lines in the second column show the observed signal-respond rates (RR) as a function of SSD. The gray violin plots show the distribution of the $1,000$ predicted response rates on each SSD. The black boxplot in each violin plot ranges from the $25^{th}$ to the $75^{th}$ percentile of the predictions. The black solid lines connect the median of the predictions across the SSDs. The dashed-dotted and dotted horizontal lines show the 95% credible interval and the median of the posterior distribution of the participant-specific $P(TF)$ parameter, respectively. The posterior predictive $p$ values on the six levels of SSDs were 0.11, 0.10, 0.37, 0.65, 0.33, and 0.35

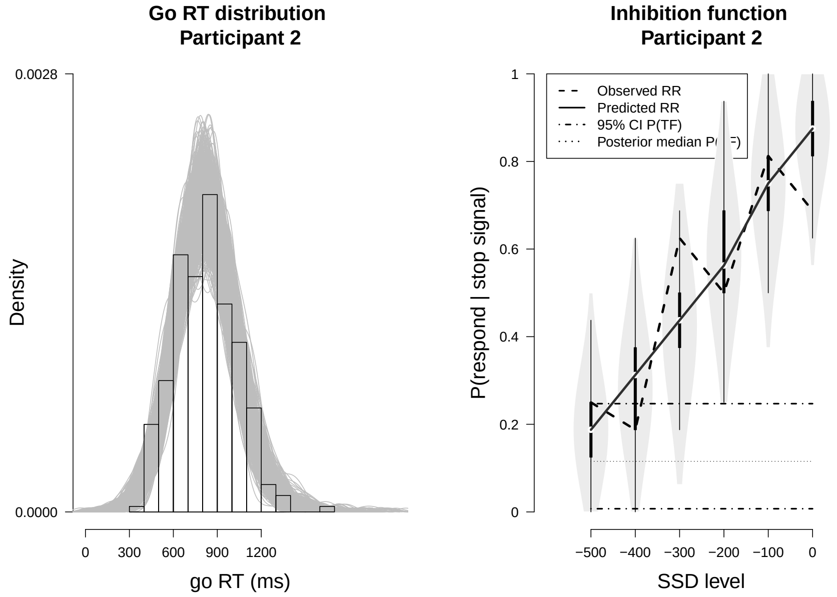


*Figure 19.* *Posterior predictive model checks for Participant* 2 *in the Badcock et al. (2002) data set.* The posterior predictive $p$ values on the six levels of SSDs were 0.15, 0.74, 0.02, 0.71, 0.20, and 0.94.

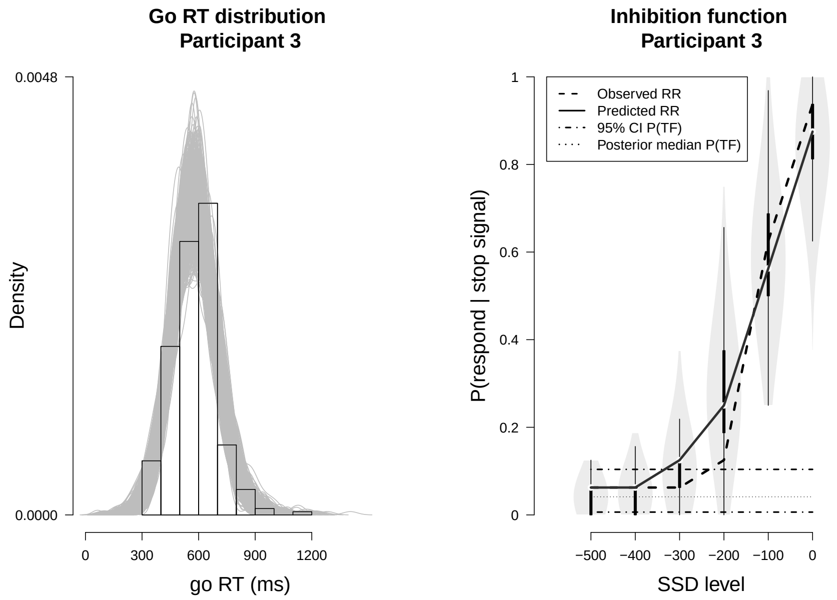


*Figure 20.* *Posterior predictive model checks for Participant* 3 *in the Badcock et al. (2002) data set.* The posterior predictive $p$ values on the six levels of SSDs were 0.04, 0.11, 0.52, 0.85, 0.30, and 0.07.

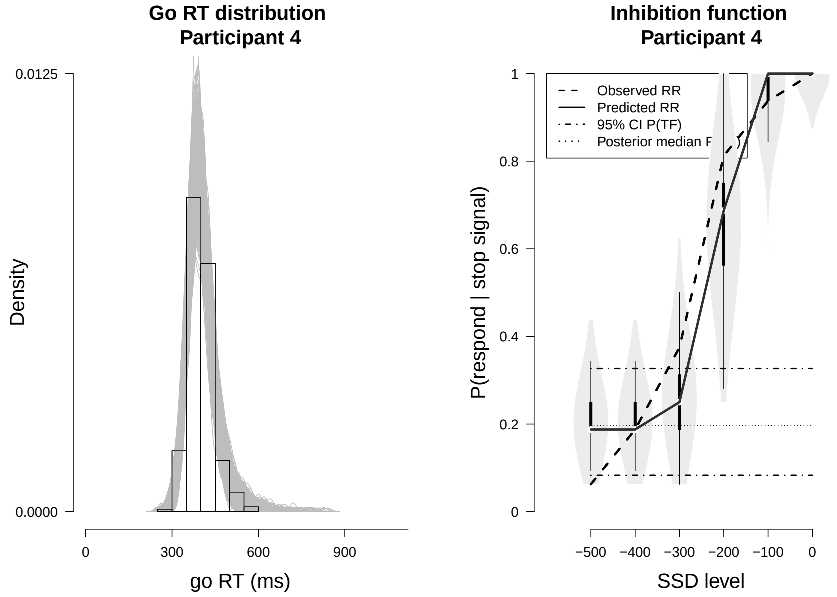


*Figure 21.* *Posterior predictive model checks for Participant* 4 *in the Badcock et al. (2002) data set.* The posterior predictive $p$ values on the six levels of SSDs were 0.97, 0.39, 0.09, 0.09, 0.55, and 0.95.

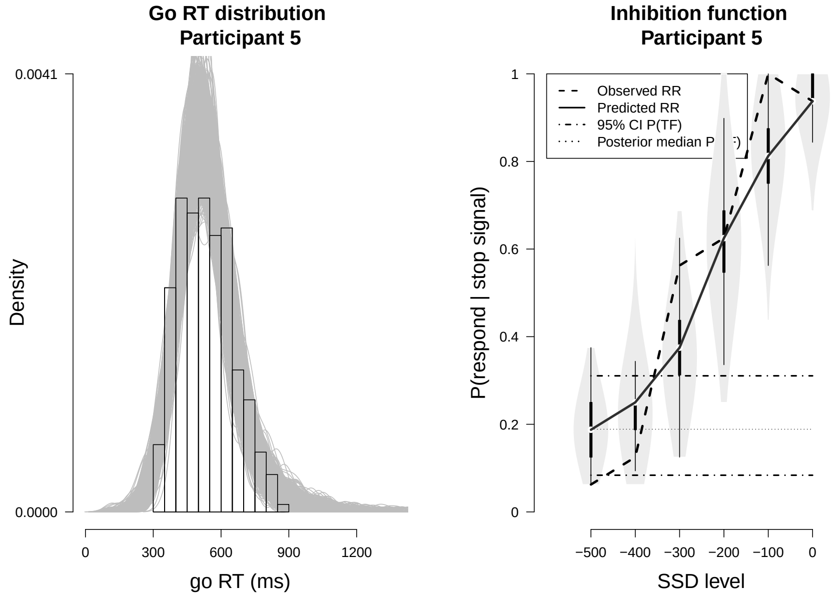


*Figure 22.* *Posterior predictive model checks for Participant* 5 *in the Badcock et al. (2002) data set.* The posterior predictive $p$ values on the six levels of SSDs were 0.98, 0.84, 0.01, 0.39, 0.06, and 0.39.

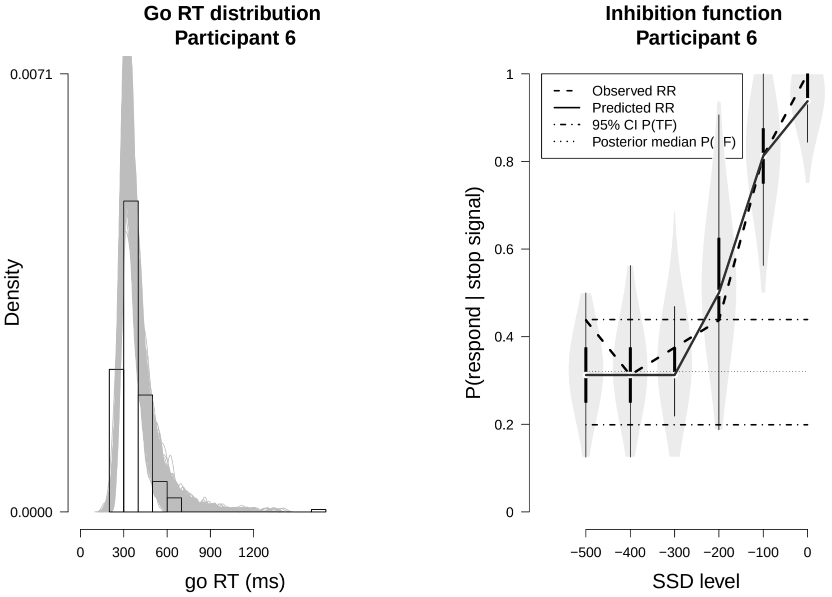


*Figure 23.* *Posterior predictive model checks for Participant* 6 *in the Badcock et al. (2002) data set.* The posterior predictive $p$ values on the six levels of SSDs were 0.01, 0.40, 0.15, 0.66, 0.44, and 0.38.

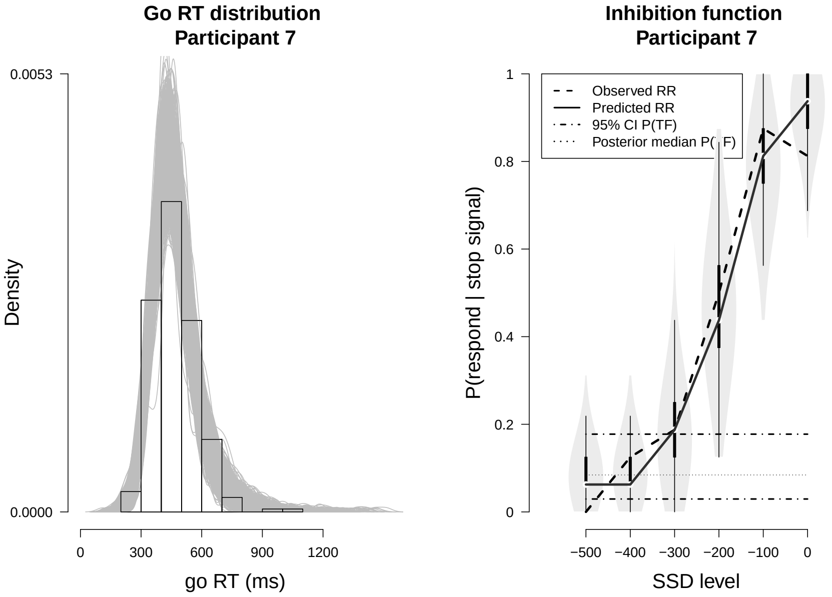


*Figure 24.* *Posterior predictive model checks for Participant* 7 *in the Badcock et al. (2002) data set.* The posterior predictive $p$ values on the six levels of SSDs were 0.96, 0.07, 0.26, 0.32, 0.12, and 0.88.

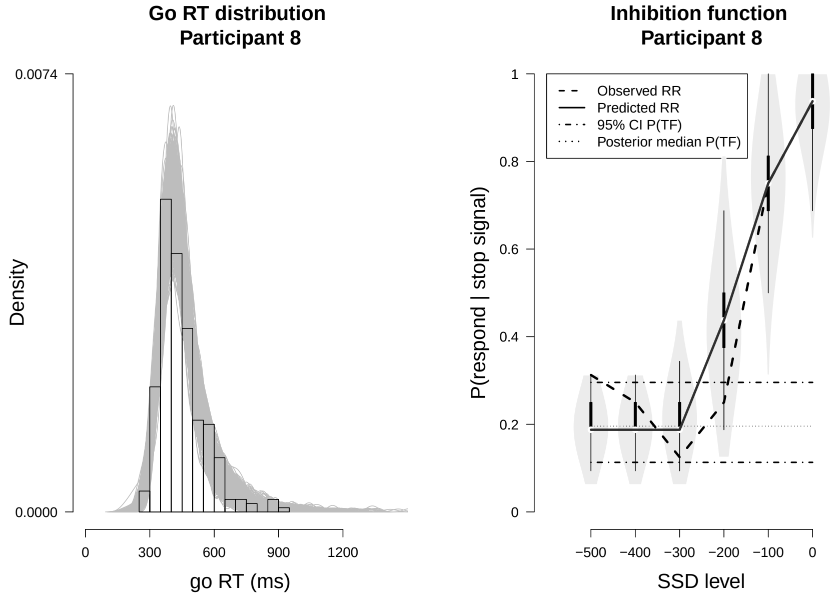


*Figure 25.* *Posterior predictive model checks for Participant* 8 *in the Badcock et al. (2002) data set.* The posterior predictive $p$ values on the six levels of SSDs were 0.01, 0.07, 0.86, 0.89, 0.47, and 0.29.

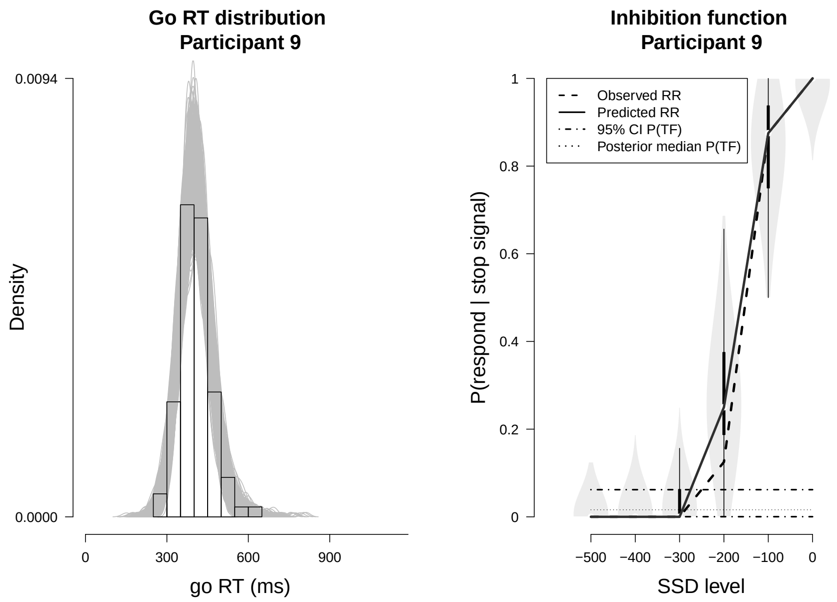


*Figure 26.* *Posterior predictive model checks for Participant* 9 *in the Badcock et al. (2002) data set.* The posterior predictive $p$ values on the six levels of SSDs were 0.16, 0.17, 0.34, 0.84, 0.27, and 0.86.

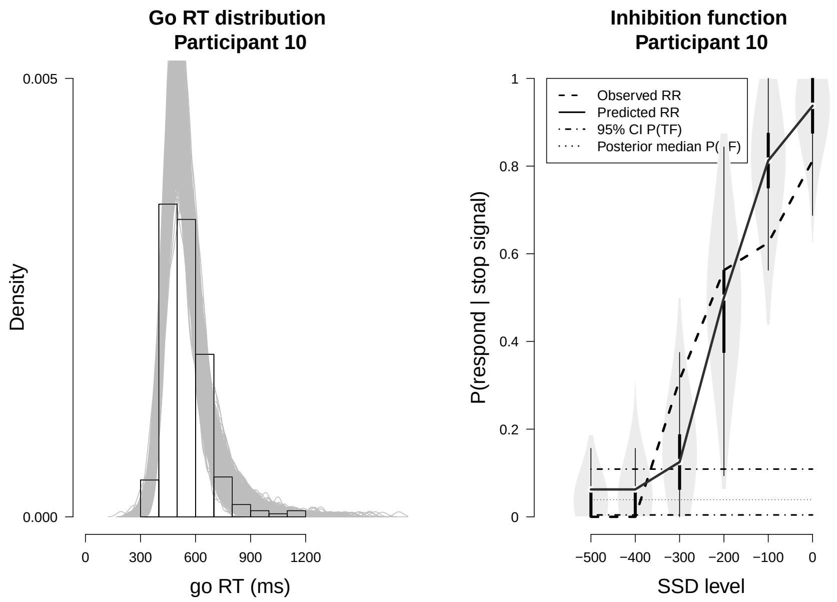


*Figure 27.* *Posterior predictive model checks for Participant* 10 *in the Badcock et al. (2002) data set.* The posterior predictive $p$ values on the six levels of SSDs were 0.54, 0.63, 0.03, 0.26, 0.89, and 0.89.

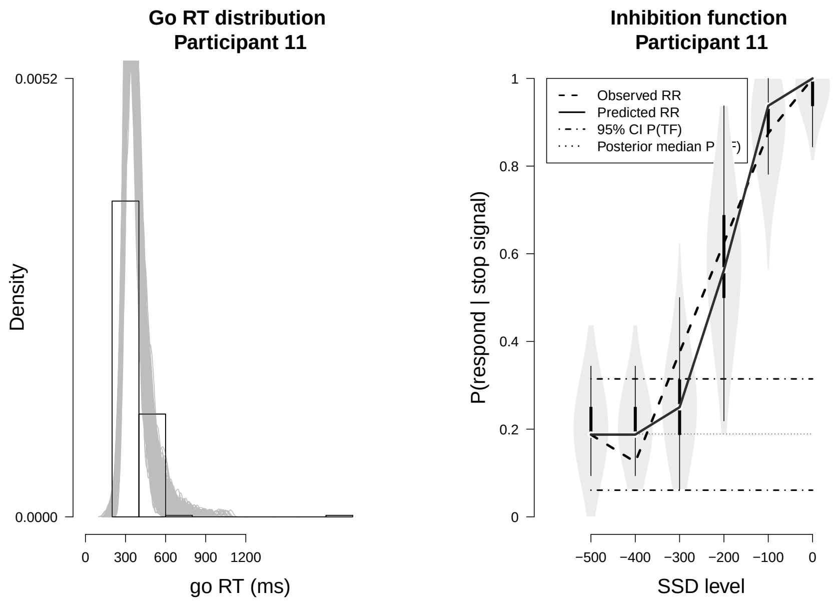


*Figure 28.* *Posterior predictive model checks for Participant* 11 *in the Badcock et al. (2002) data set.* The posterior predictive $p$ values on the six levels of SSDs were 0.38, 0.78, 0.06, 0.31, 0.49, and 0.68.

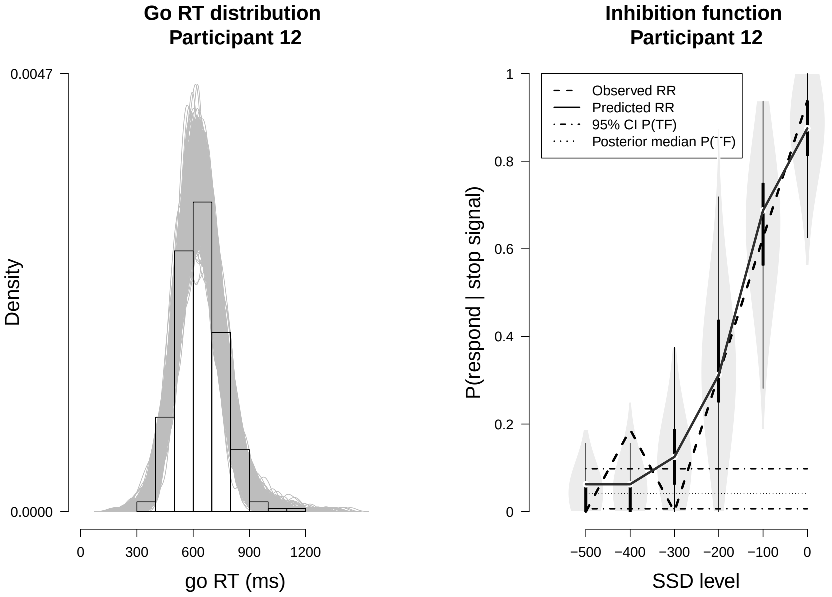


*Figure 29.* *Posterior predictive model checks for Participant* 12 *in the Badcock et al. (2002) data set.* The posterior predictive $p$ values on the six levels of SSDs were 0.61, 0.00, 0.88, 0.41, 0.49, and 0.15.

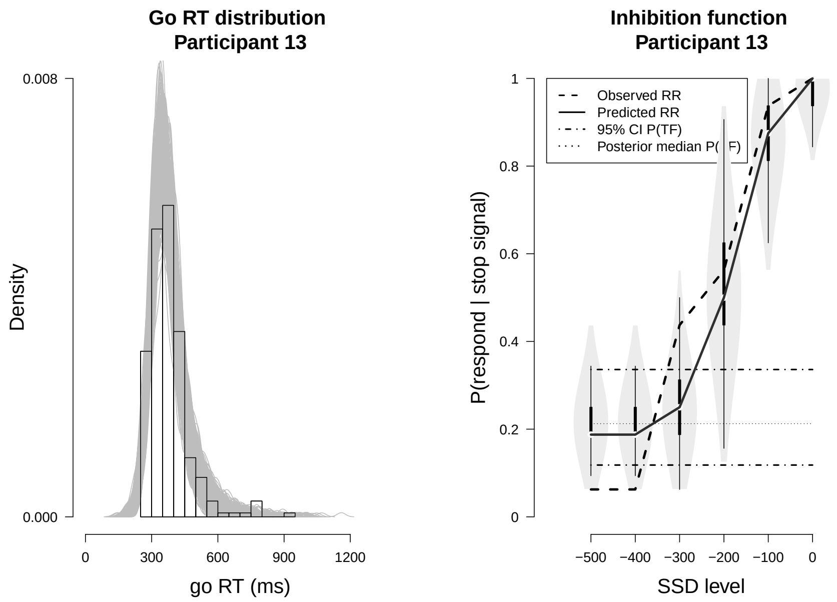


*Figure 30.* *Posterior predictive model checks for Participant* 13 *in the Badcock et al. (2002) data set.* The posterior predictive $p$ values on the six levels of SSDs were 1.00, 1.00, 0.01, 0.26, 0.10, and 0.62.

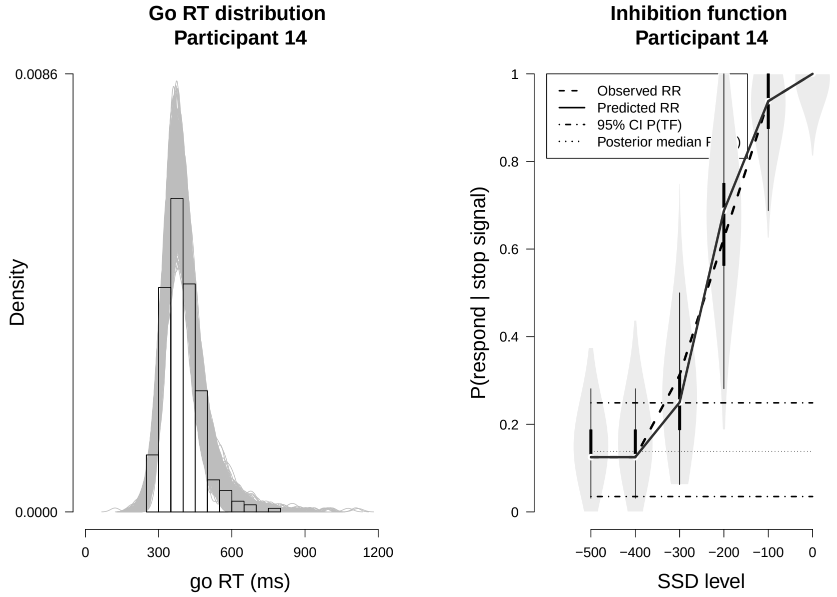


*Figure 31.* *Posterior predictive model checks for Participant* 14 *in the Badcock et al. (2002) data set.* The posterior predictive $p$ values on the six levels of SSDs were 0.45, 0.50, 0.25, 0.55, 0.34, and 0.83.

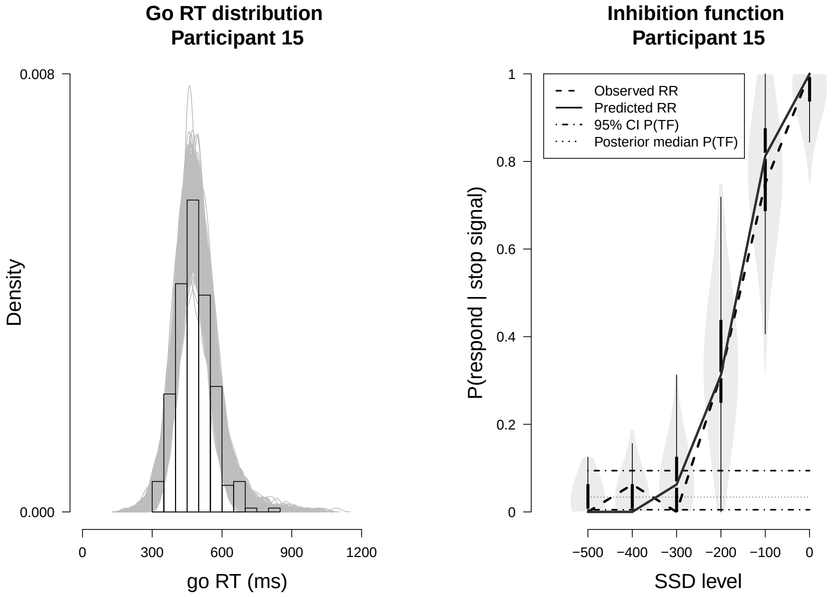


*Figure 32.* *Posterior predictive model checks for Participant* 15 *in the Badcock et al. (2002) data set.* The posterior predictive $p$ values on the six levels of SSDs were 0.47, 0.03, 0.71, 0.47, 0.50, and 0.60.

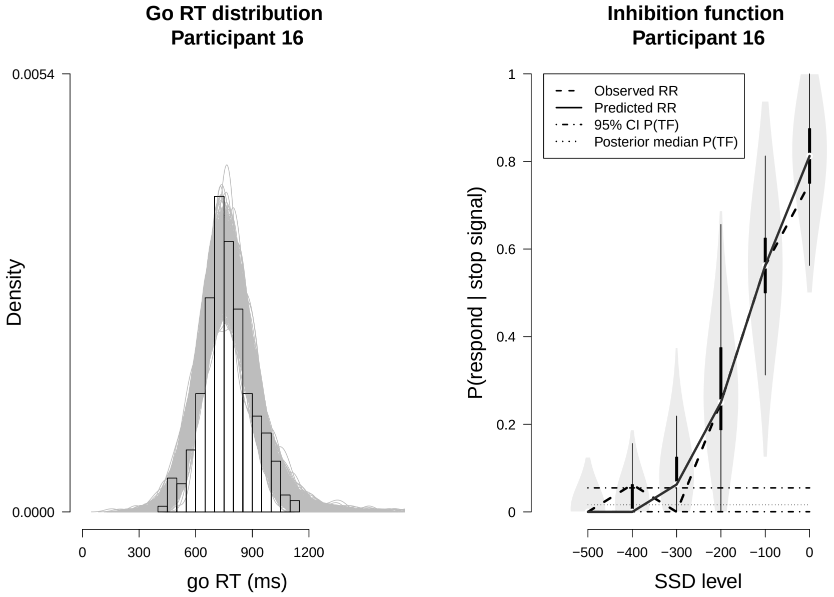


*Figure 33.* *Posterior predictive model checks for Participant* 16 *in the Badcock et al. (2002) data set.* The posterior predictive $p$ values on the six levels of SSDs were 0.17, 0.04, 0.77, 0.47, 0.40, and 0.62.

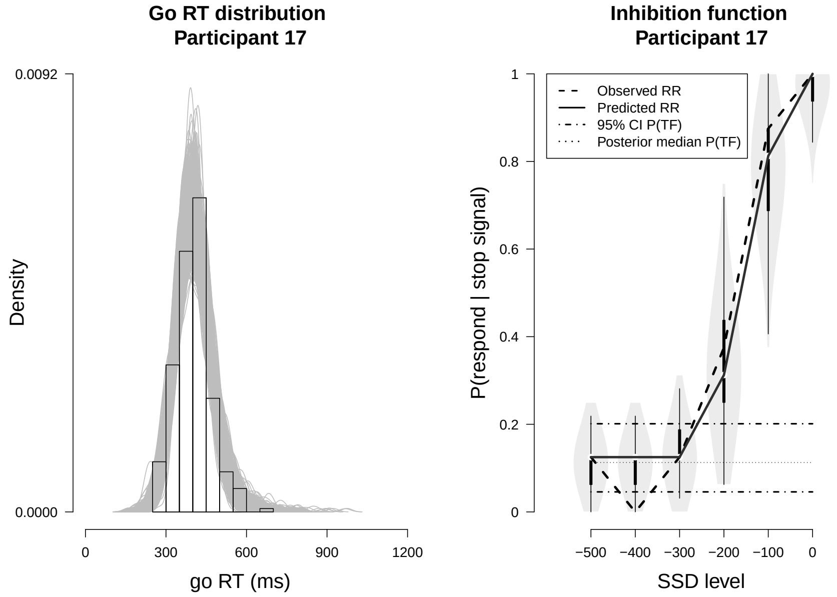


*Figure 34.* *Posterior predictive model checks for Participant* 17 *in the Badcock et al. (2002) data set.* The posterior predictive $p$ values on the six levels of SSDs were 0.14, 1.00, 0.26, 0.25, 0.15, and 0.69.

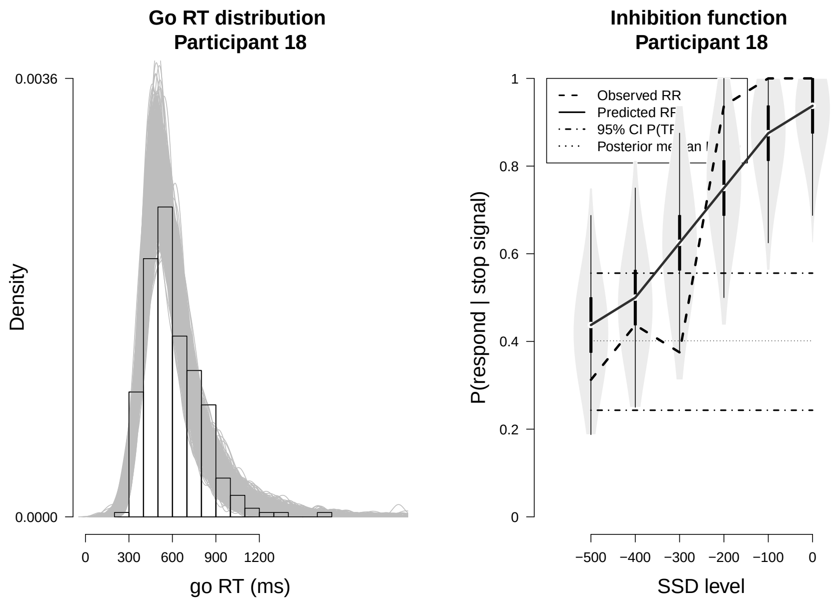


*Figure 35.* *Posterior predictive model checks for Participant* 18 *in the Badcock et al. (2002) data set.* The posterior predictive $p$ values on the six levels of SSDs were 0.82, 0.57, 0.99, 0.02, 0.10, and 0.29.

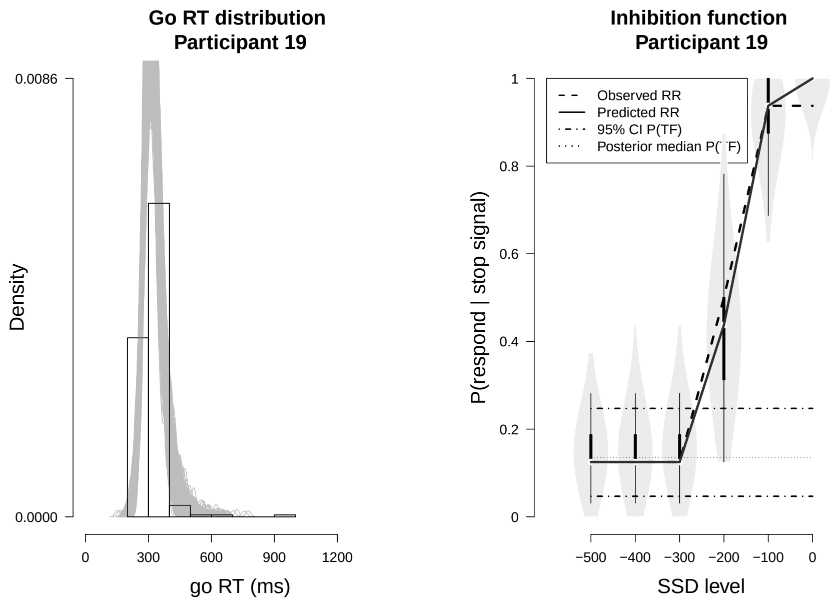


*Figure 36.* *Posterior predictive model checks for Participant* 19 *in the Badcock et al. (2002) data set.* The posterior predictive $p$ values on the six levels of SSDs were 0.43, 0.44, 0.49, 0.21, 0.23, and 0.90.

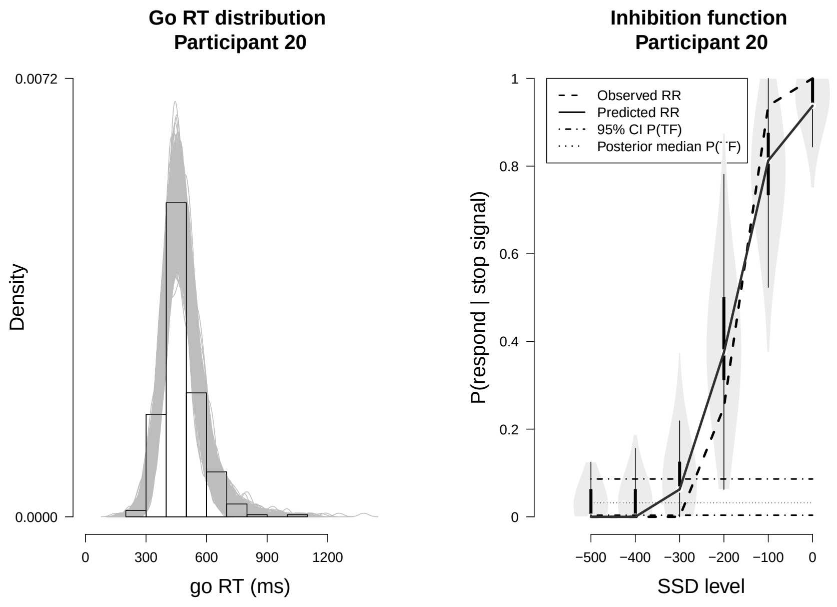


*Figure 37.* *Posterior predictive model checks for Participant* 20 *in the Badcock et al. (2002) data set.* The posterior predictive $p$ values on the six levels of SSDs were 0.45, 0.47, 0.78, 0.77, 0.02, and 0.47.

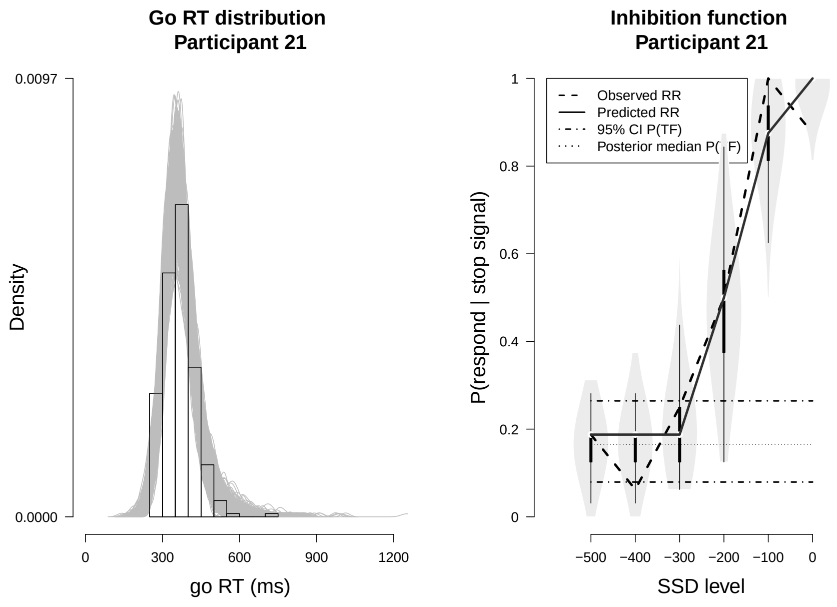


*Figure 38.* *Posterior predictive model checks for Participant* 21 *in the Badcock et al. (2002) data set.* The posterior predictive $p$ values on the six levels of SSDs were 0.16, 0.96, 0.08, 0.35, 0.17, and 0.97.

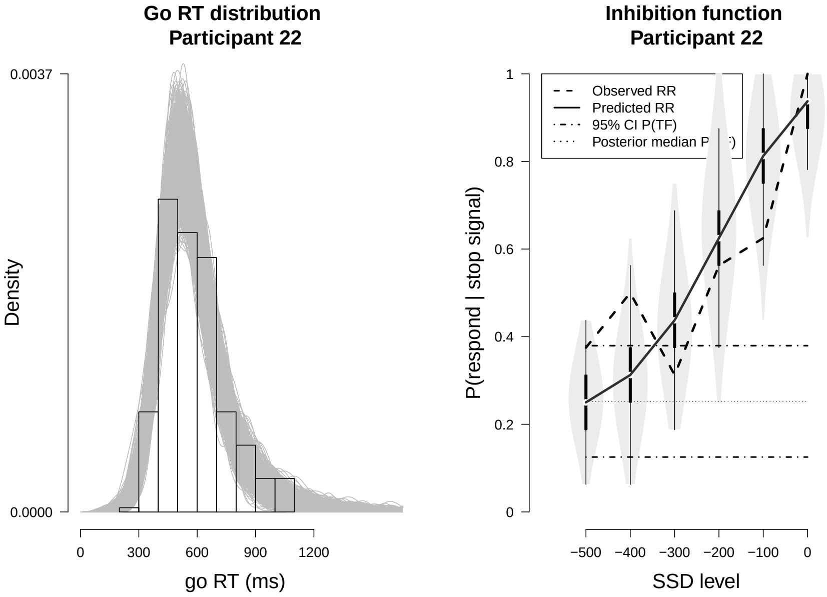


*Figure 39.* *Posterior predictive model checks for Participant* 22 *in the Badcock et al. (2002) data set.* The posterior predictive $p$ values on the six levels of SSDs were 0.02, 0.00, 0.78, 0.66, 0.92, and 0.21.

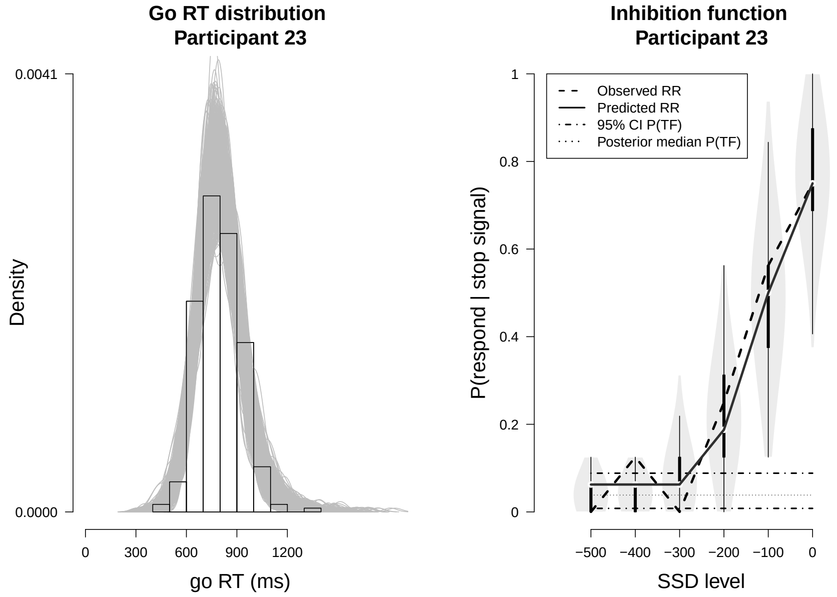


*Figure 40.* *Posterior predictive model checks for Participant* 23 *in the Badcock et al. (2002) data set.* The posterior predictive $p$ values on the six levels of SSDs were 0.56, 0.00, 0.77, 0.27, 0.24, and 0.47.

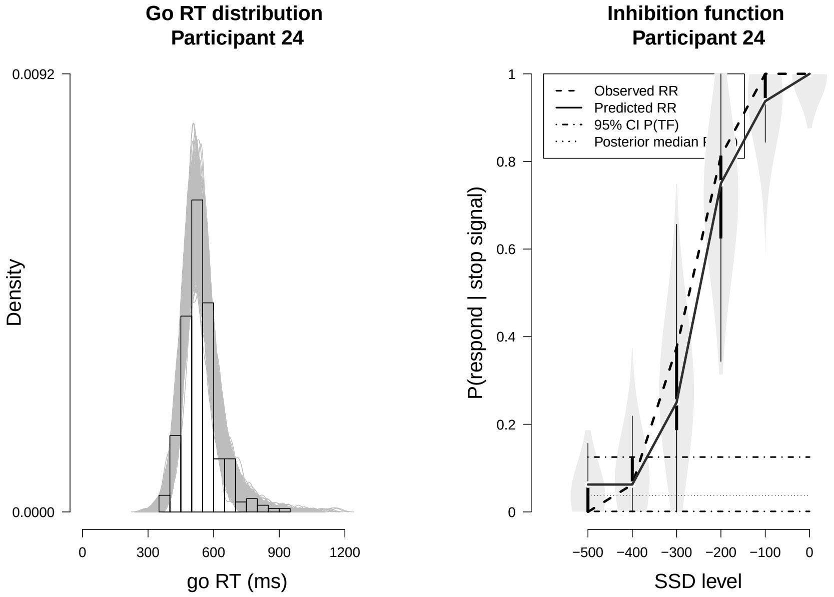


*Figure 41.* *Posterior predictive model checks for Participant* 24 *in the Badcock et al. (2002) data set.* The posterior predictive $p$ values on the six levels of SSDs were 0.55, 0.36, 0.17, 0.17, 0.45, and 0.86.

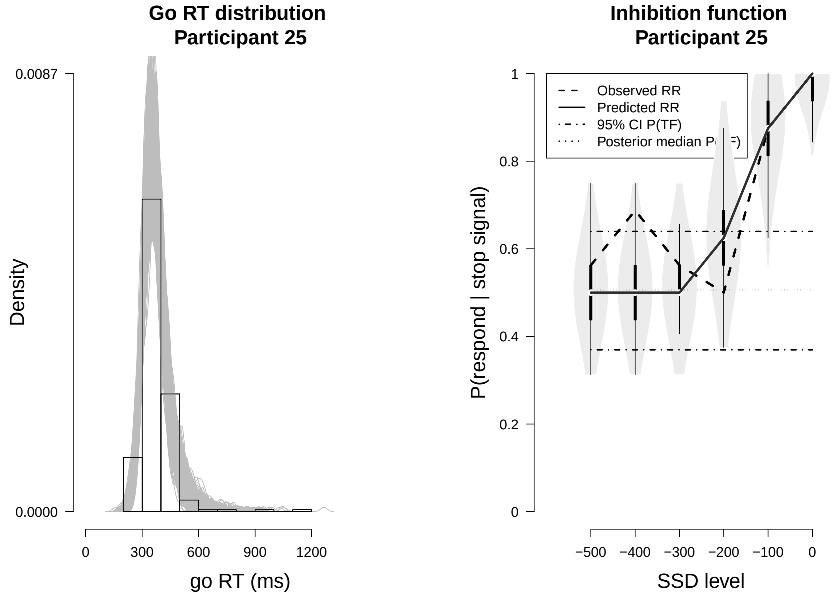


*Figure 42. Posterior predictive model checks for Participant* 25 *in the Badcock et al. (2002) data set.* The posterior predictive $p$ values on the six levels of SSDs were 0.09, 0.00, 0.12, 0.88, 0.46, and 0.72.

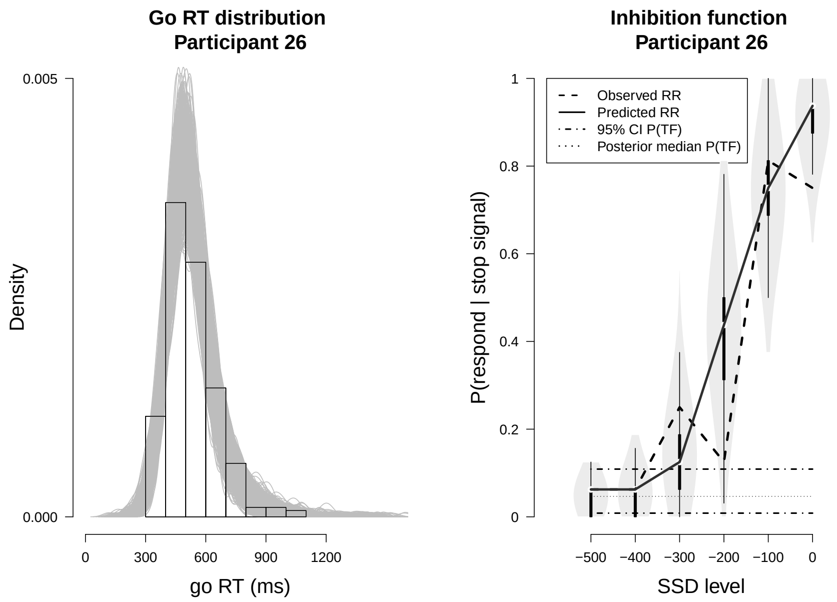


*Figure 43.* *Posterior predictive model checks for Participant* 26 *in the Badcock et al. (2002) data set.* The posterior predictive $p$ values on the six levels of SSDs were 0.05, 0.13, 0.06, 0.99, 0.23, and 0.95.

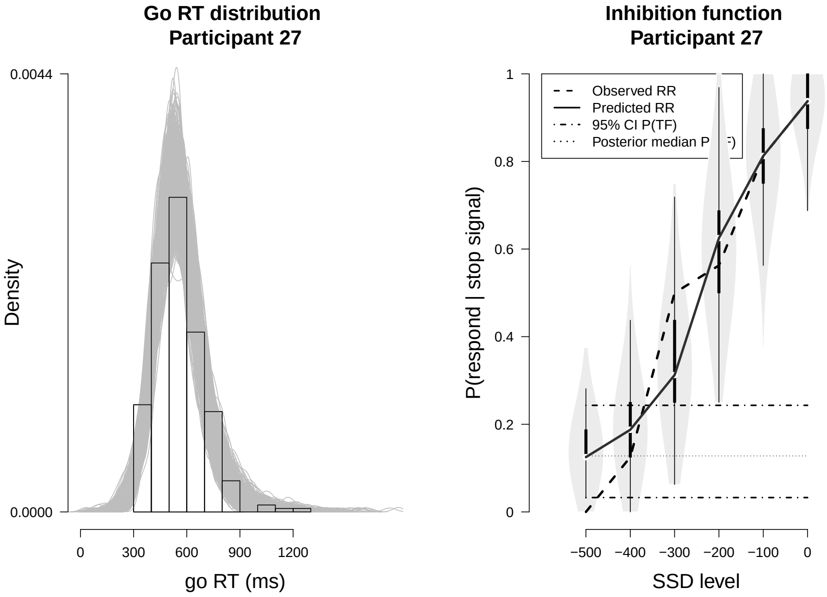


*Figure 44.* *Posterior predictive model checks for Participant* 27 *in the Badcock et al. (2002) data set.* The posterior predictive $p$ values on the six levels of SSDs were 0.98, 0.66, 0.05, 0.50, 0.43, and 0.33.

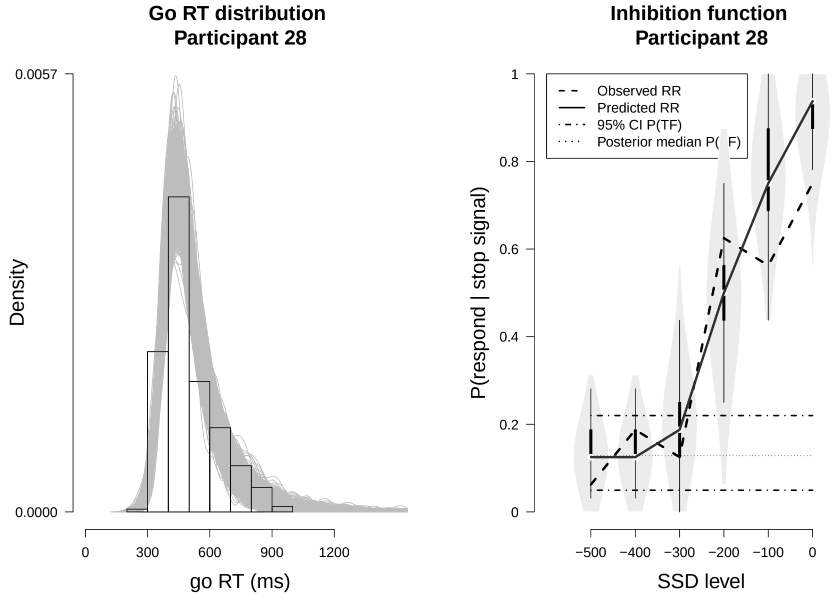


*Figure 45.* *Posterior predictive model checks for Participant* 28 *in the Badcock et al. (2002) data set.* The posterior predictive $p$ values on the six levels of SSDs were 0.77, 0.05, 0.70, 0.10, 0.94, and 0.91.

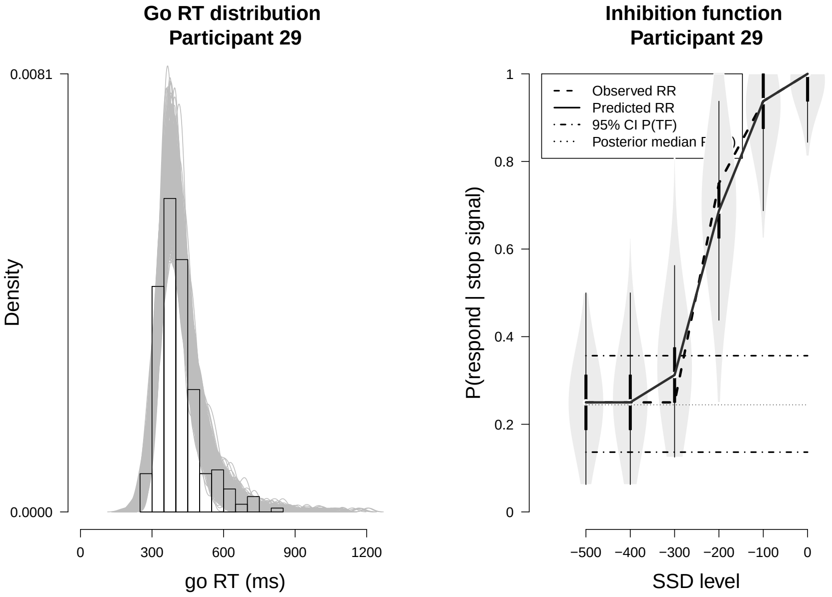


*Figure 46.* *Posterior predictive model checks for Participant* 29 *in the Badcock et al. (2002) data set.* The posterior predictive $p$ values on the six levels of SSDs were 0.32, 0.36, 0.74, 0.27, 0.26, and 0.70.

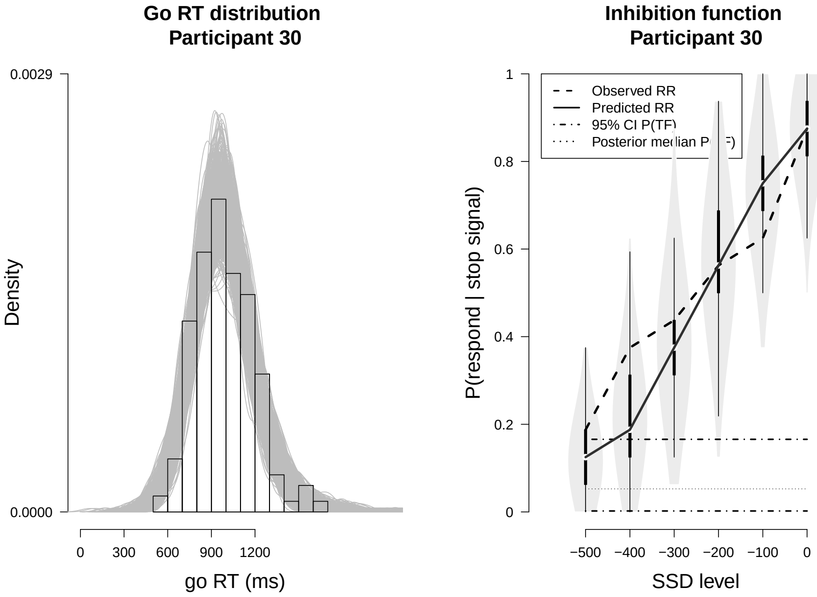


*Figure 47.* *Posterior predictive model checks for Participant* 30 *in the Badcock et al. (2002) data set.* The posterior predictive $p$ values on the six levels of SSDs were 0.13, 0.05, 0.25, 0.46, 0.78, and 0.35.

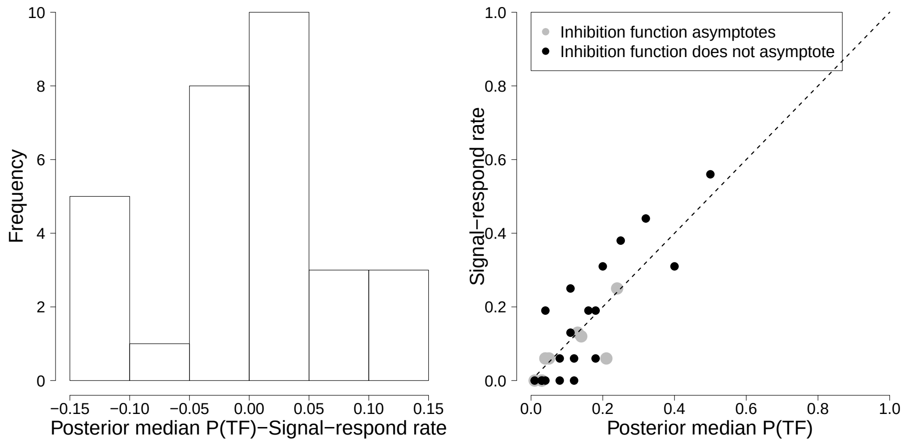


*Figure 48.* *The relationship between the participant-specific $P(TF)$ parameters and the observed signal-respond rate at the shortest SSD.* The left panel shows the distribution of the differences between the posterior median of the participant-specific $P(TF)$ parameters and signal-respond rate at the shortest SSD for the 30 participants. The right panel shows a scatterplot between the posterior median of the participant-specific $P(TF)$ parameters and signal-respond rate at the shortest SSD. The black bullets show participants with no clear lower asymptote in the inhibition function. The gray bullets show participants with a clear lower asymptote in the inhibition function; for these participants, the $P(TF)$ parameter very closely approximated the lower asymptote of the observed inhibition function.

# References

Badcock, J. C., Michie, P., Johnson, L., & Combrinck, J. (2002). Acts of control in schizophrenia: Dissociating the components of inhibition. *Psychological Medicine*, *32*, 287–297.

Hughes, M. E., Fulham, W. R., Johnston, P. J., & Michie, P. T. (2012). Stop-signal response inhibition in schizophrenia: Behavioural, event-related potential and functional neuroimaging data. *Biological Psychology*, *89*, 220–231.
